# High-throughput single-cell isolation of *Bifidobacterium* strains from the human gut microbiome

**DOI:** 10.1128/spectrum.03033-25

**Published:** 2025-12-30

**Authors:** Lam Hai Ha, Yue Yuan On, Clarice Pohan, Jungwon Lee, Shaun Hong Chuen How, Yik-Ying Teo, Henning Seedorf, Jean-Sebastien Gounot, Niranjan Nagarajan

**Affiliations:** 1 Genome Institute of Singapore (GIS), Agency for Science, Technology and Research (A*STAR) 68695, Singapore, Singapore; 2 Life Sciences Institute, National University of Singapore 37580, Singapore, Singapore; 3 Saw Swee Hock School of Public Health, National University of Singapore 37580, Singapore, Singapore; 4 Department of Statistics and Applied Probability, National University of Singapore 37580, Singapore, Singapore; 5 Temasek Lifesciences Laboratory, Singapore, Singapore; 6 Department of Biological Sciences, National University of Singapore 37580, Singapore, Singapore; 7 Department of Biochemistry, Yong Loo Lin School of Medicine, National University of Singapore 37580, Singapore, Singapore; Quest Diagnostics Nichols Institute, Chantilly, Virginia, USA

**Keywords:** high-throughput isolation, culturomics, *Bifidobacterium*

## Abstract

*Bifidobacterium* represents a diverse genus of commensal gut bacteria with key roles in human health, from metabolizing indigestible fibers to protecting against pathogens. While metagenomic studies have highlighted significant strain diversity for *Bifidobacterium* species within individuals, their systematic isolation and phenotypic characterization can be hampered by the significant effort and biases inherent in traditional culturomics. Here, we explored the utility of a high-throughput single-cell dispensing system (B.SIGHT)-based workflow for accelerating the process of isolating diverse *Bifidobacterium* strains from fecal samples. Systematic assessment of this workflow revealed a high single-cell dispensing frequency (>88%) and the ability to preserve species diversity when a pool of *Bifidobacterium* strains was dispensed. Culturing-related factors including the use of an effective selection medium, such as the Bifidus Selective Medium supplemented with mupirocin, and the length of pre-dispensing incubation were found to be critical in determining isolation success. Leveraging this workflow, we obtained a total of 622 viable isolates from five Singaporean fecal samples, of which >98% were found to be from *Bifidobacterium* species. Whole-genome sequencing of 96 isolates identified six different *Bifidobacterium* species with both inter- and intra-subject strain and lineage diversity, and the majority (>66%) were novel relative to large public genomic databases. Our findings highlight the ability of this high-throughput culturomics workflow to accelerate the recovery of diverse and novel *Bifidobacterium* strains, enabling further interrogation of their functional characteristics and advancing our understanding of important bacterial species in the gut microbiome.

**IMPORTANCE:**

The field of high-throughput microbial culturomics is still in its early stages. Enhancing our ability to isolate and phenotypically test bacterial strains from complex communities is crucial for advancing microbiome research and healthcare development. Given the time and cost inefficiencies of traditional culturing methods, a more efficient, high-throughput approach to obtain isolates is needed. In the present study, we assessed a single-cell dispensing platform and developed a workflow to isolate diverse *Bifidobacterium* strains from fecal samples. We demonstrated here the capability of this novel technology to efficiently obtain hundreds of isolates of a targeted group, covering both species and strain diversities. This generalizable and scalable method can potentially allow for the high-throughput recovery of microbes from other taxonomic groups, providing a fundamental step in improving the culturomics framework to complement metagenomic approaches and enable isolate-level functional studies of important microbes.

## INTRODUCTION

Culturomics is an essential tool for understanding the diversity of microbial communities (1, 2). By leveraging different culturing media and conditions, it has been successfully used to cultivate bacteria from several environments, such as plant (3, 4), soil (5–7), and human microbiota (8–11), identifying previously unknown bacteria and thereby filling the microbial “dark matter” gap highlighted by metagenomic studies (8, 12–16). Another advantage of culturomics is its ability to enable better characterization of bacteria both phenotypically and genotypically. Culturomics yields live isolates that enable direct assessment of microbial phenotypes such as metabolic profiles, antibiotic resistance, or host-environment interactions that cannot be definitively established through genomic information alone (17–20). Additionally, the ability to deeply sequence individual isolates results in more accurate and complete genome assembly, especially for low-abundance microbes in a community (21–23). Such high-resolution genomic data can then enable the discovery of novel species and strain-level variations, including person- and population-specific signatures.

Despite its utility, the application of culturomics remains limited by time-consuming, labor-intensive, and low-throughput bacterial isolation methods (24, 25). Thus, to increase the recovery efficiency of culturomics, automated single-cell isolation technologies have been progressively developed. Notably, microfluidic and fluorescence-activated cell sorting (FACS) systems were among the first to be used. While microfluidic systems are more affordable and physically compact, they lack control over the end products, resulting in large numbers of empty droplets (26, 27). On the other hand, FACS systems can achieve high single-cell recovery rates but can be demanding in terms of expertise and cost of setup (28, 29). In addition, FACS systems require a large amount of space, which makes them incompatible with enclosed environments such as an anaerobic chamber. Recently, next-generation single-cell dispensers have offered an alternative solution that provides a more custom and compact system for high-throughput single-cell isolation (24, 27, 30), but with limited information about their capabilities and correspondingly development of workflows that leverage them.

In this study, we first characterized the performance of one such dispensing technology (B.SIGHT, Cytena GmBH, Germany) in terms of its doublet frequency, validating its utility as a single-cell isolation platform. We then applied the single-cell dispenser in a culturomics workflow to efficiently isolate *Bifidobacterium* species from fecal samples. We chose *Bifidobacterium* as our model bacterial genus as it plays many important roles in the human gut (31–35) and, in a recent metagenomic study from our group, significant uncharacterized *Bifidobacterium* diversity was highlighted in Singaporean gut microbiomes (36). To understand the dispenser’s behavior when handling a mixed bacterial community, we dispensed nine *Bifidobacterium* species at once and found that the single-cell dispenser was able to capture the species-level diversity of this mock community. We also demonstrate that the pre-dispensing incubation period and choice of enrichment media play a key role in determining success in recovering diverse *Bifidobacterium* species. Finally, we successfully dispensed a large collection of *Bifidobacterium* isolates from five Singaporean fecal samples, identifying substantial strain and lineage-level diversity. This study serves as a proof of concept for the application of single-cell dispensers in culturomics workflows, paving the way for larger population-scale studies involving isolation of microbial strains from diverse microbiomes.

## RESULTS

### Characterization of a single-cell dispensing system’s performance in isolating pure bacterial cultures

#### Assessment of single-cell dispensing frequency and possibility of cartridge reuse

**Fig 1:**
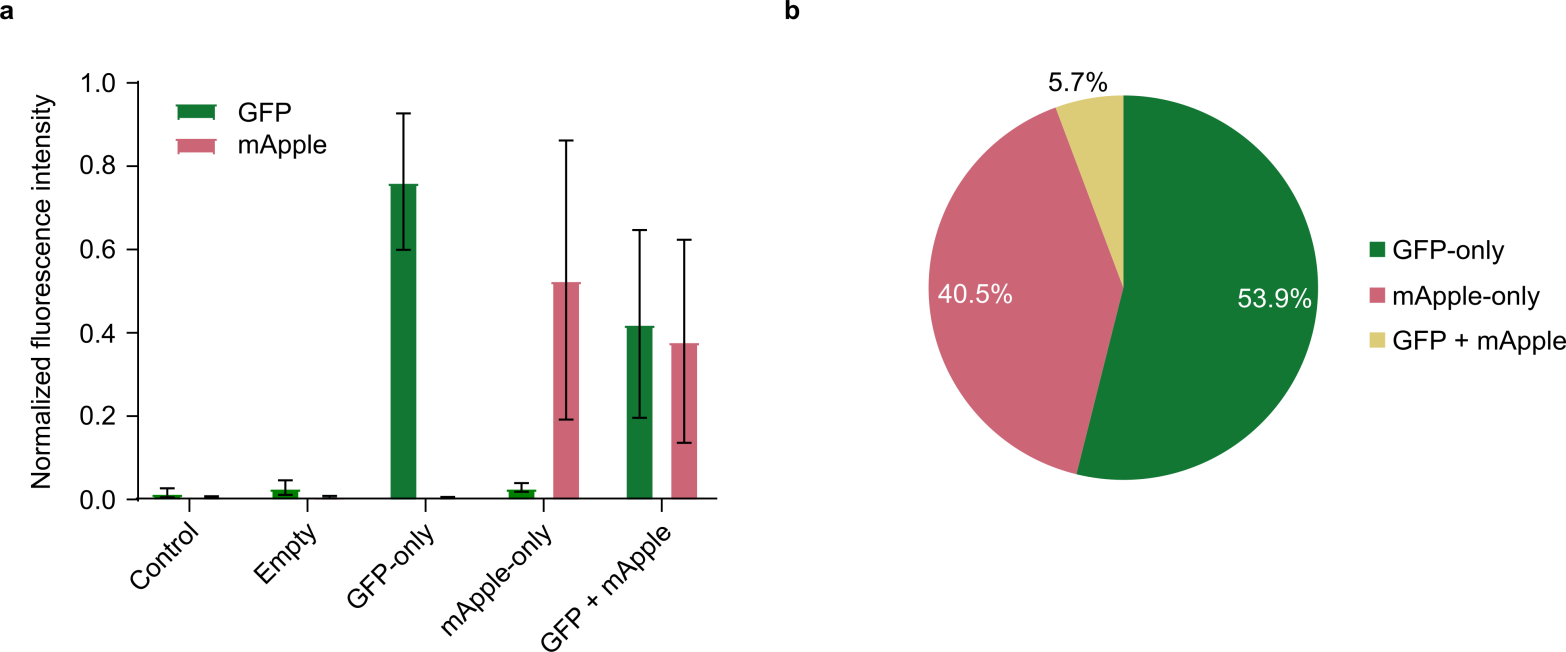
Fluorescence signals detected from individual wells when dispensing a mixed culture of GFP-expressing and mApple-expressing *K. pneumoniae*. (**a**) Normalized fluorescence intensity detected from individual wells after dispensing of a mixed GFP-expressing and mApple-expressing *K. pneumoniae* culture (*Control:* control wells where no cells were dispensed; *Empty:* wells with no signals for bacterial culture). A positive fluorescence signal threshold was defined as two standard deviations from the mean background fluorescence of control wells. (**b**) Proportions of wells that showed only GFP, only mApple, or both signals.

To validate the single-cell isolation capability of the B.SIGHT system, we dispensed a mixed culture of green fluorescent protein (GFP)-expressing and mApple-expressing *K. pneumoniae* in roughly equal proportions into six 96-well plates and detected fluorescence signals from each individual well. A majority of non-empty wells (450/477 or 94.3%, Table S1) displayed a single fluorescence signal (Fig. 1a, GFP- and mApple-only), while a group of wells (27/477, Table S1) showed both GFP and mApple signals (Fig. 1a, GFP + mApple). This result suggests that approximately 5.66% of the dispensed wells contained at least two cells with different fluorescence signals (Fig. 1b). Given the observed proportions of GFP-only, mApple-only, and GFP + mApple wells, we estimate that 11.5% of the wells contained at least two cells, including those that present as a single fluorescence signal. Thus, here we found an estimated overall single-cell dispensing frequency of >88%, indicating that the system is reliable for enriching for isolates from a mixed sample, though additional re-streaking may be needed for higher purity. We also tested the reusability of the dispensing cartridge to reduce operational costs for future experiments. By introducing a post-dispensing washing procedure to the dispensing workflow, the number of cells detected in the cartridge approaches near-zero levels for samples with varying cell densities (Fig. S1a). Specifically, after three phosphate-buffered saline (PBS) washes, we achieved a negligible (~0.1%) contamination rate for medium- to high-density samples and a ~2% rate for low-density samples, which can be further reduced to ~0.4% upon two additional washes (Fig. S1b). This washing procedure further improves the cost efficiency and utility of the single-cell dispenser in processing a large number of samples (each cartridge costs >US$350).

#### A single-cell dispenser-based workflow captures species-level diversity in mixed *Bifidobacterium* samples

**Fig 2:**
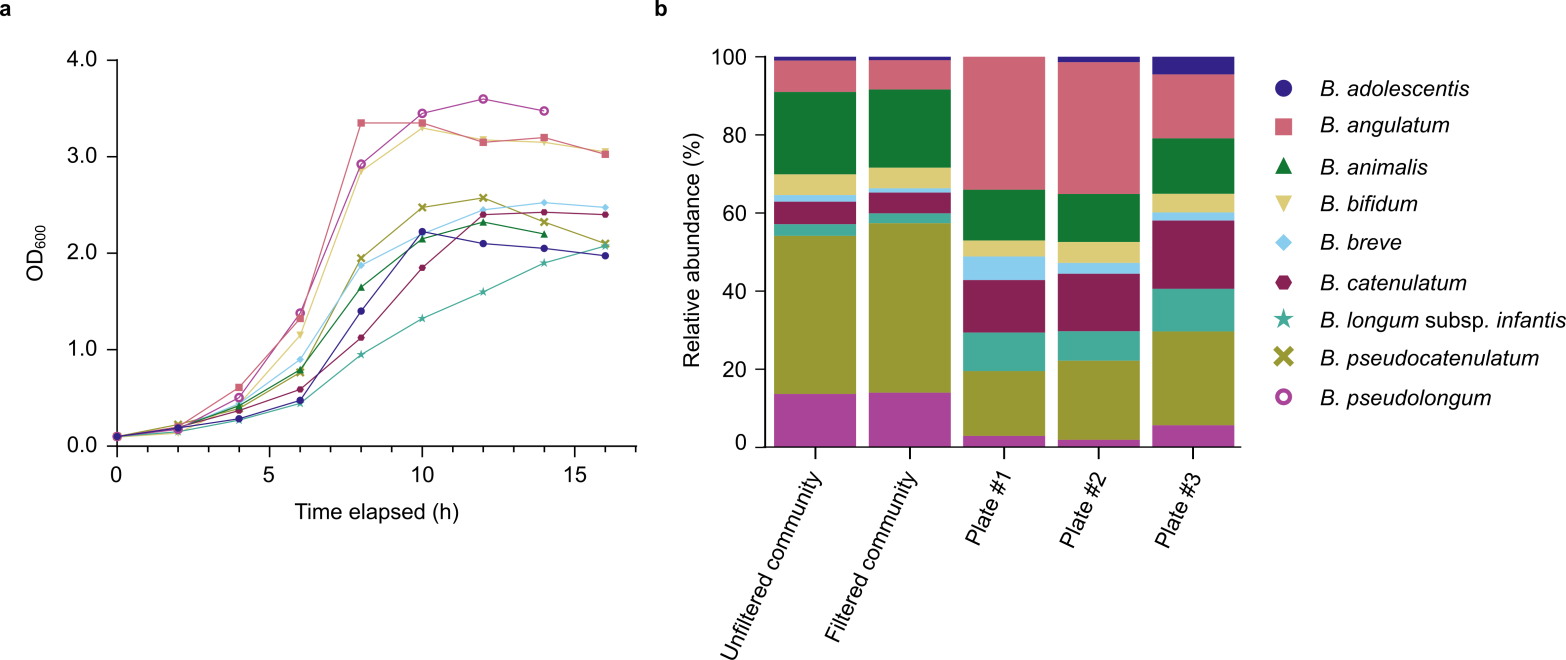
Growth curves and post-dispensing changes in relative abundances of nine *Bifidobacterium* species in a mock community. (**a**) Growth curves of nine *Bifidobacterium* species based on monitoring OD_600_ over a 16 h period. (**b**) Relative abundances of *Bifidobacterium* species from the mock community before and after dispensing, as determined by shotgun sequencing and taxonomic profiling (*Unfiltered community*: mock community before filtering with a 20 µm cell strainer; *Filtered community*: mock community after filtering with a 20 µm cell strainer).

To investigate whether single-cell dispensing can preserve the global bacterial diversity of samples, we dispensed a mock community of nine *Bifidobacterium* species. To obtain the mock community, the *Bifidobacterium* species were cultured separately until their mid-log phase, as determined from their individual growth curves (Fig. 2a) in Bifidus Selective Medium (BSM), a highly selective medium that supports the growth of *Bifidobacterium* while inhibiting that of other genera such as *Lactobacillus*, *Streptococcus*, *Enterococcus,* and molds. We supplemented the medium with lithium mupirocin, an antibiotic commonly used as a selective agent in *Bifidobacterium* isolation with high reported specificity (referred to here as “BSM-MUP”). Of note, three of the *Bifidobacterium* species (*B. angulatum*, *B. pseudolongum,* and *B. bifidum*) showed slightly faster growth rates compared to the rest. The nine *Bifidobacterium* species were pooled to form a mock community and passed through a 20 μm strainer to remove large cell clumps prior to dispensing into BSM-MUP. Sequencing of the mock community before and after filtering revealed similar species profiles, indicating that the filtering step did not introduce species-specific biases into the dispensing workflow (Fig. 2b). Although the initial mock community displayed uneven abundances, all nine species were recovered at >1% relative abundance in post-dispensing plates, including low-abundance members such as *B. adolescentis* (1% in filtered community), demonstrating the system’s ability to dispense all species. Nonetheless, notable shifts in the relative abundance were observed following dispensing, with *B. angulatum* exhibiting the largest increase (+19% on average, *n* = 3, *P*>0.05) and *B. pseudocatenulatum* showing the largest decrease (−25% on average, *n* = 3, *P*<0.05; Fig. S2). Some of these shifts may be partially explained by cell morphology differences, suggesting a possible bias in the dispenser’s cell recognition algorithm. For instance, *B. animalis* and *B. bifidum*, which both showed a reduction in post-dispensing abundance, are considerably more elongated in shape than other species when observed under a microscope (Fig. S3). Additionally, *B. angulatum* was uniquely prone to forming sticky clumps during the mid-log phase, which may have affected its representation during dispensing. Overall, while the single-cell dispenser may not preserve relative proportions of microbes, it shows promise as a valuable tool for capturing and isolating the global diversity of complex bacterial communities.

#### Choice of the enrichment medium and incubation period impact the culture success rate of *Bifidobacterium* species upon dispensing

**Fig 3:**
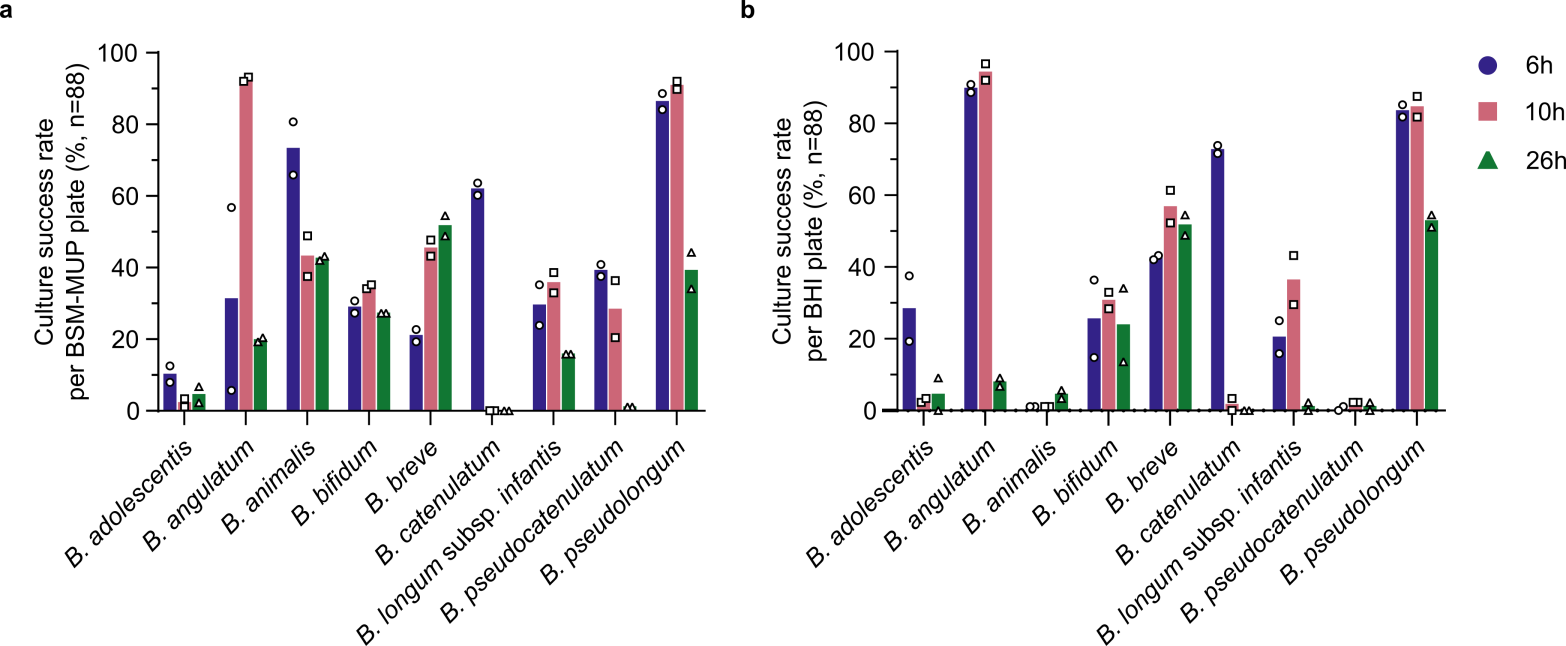
Culture success rates upon dispensing individual pure cultures of *Bifidobacterium* species at various incubation time points in BSM-MUP and BHI. (**a**) BSM-MUP and (**b**) BHI. Culture success rates were calculated as the percentage of viable cultures per 96-well plate (*n* = 88).

Having assessed the dispenser’s capabilities, we proceeded to evaluate different culturing factors that could affect the overall culture success rate. All nine *Bifidobacterium* species from the mock community were cultured separately in BSM-MUP for 6 h, 10 h, and 26 h. At each predetermined incubation timepoint, pure cultures were dispensed into duplicate plates containing either BSM-MUP or Brain Heart Infusion (BHI), a general-purpose, antibiotic-free medium. With this experimental setup, we aimed to assess whether the post-dispensing culture rate is affected by the incubation period or medium used. When comparing different incubation periods, *Bifidobacterium* species exhibited notable differences in peak culture success rates, corresponding to differences in their growth phases (Fig. 3). Considering only the BSM-MUP plates, some species such as *B. adolescentis*, *B. animalis*, and *B. catenulatum* were most successfully isolated in their early log phase (6 h), while others were better recovered in their mid- to late log phase at 10 h (Fig. 3a). At 26 h, the recovery rates were mostly lower for all species, except for *B. breve*. Given the differences observed in culture rates, we determined that varying the incubation period would be an essential component in isolating *Bifidobacterium* and obtaining higher recovery across different species. Comparing between BSM-MUP and BHI, similar optimum (across incubation periods) culture success rates were observed for seven out of the nine species (Fig. 3). However, for *B. animalis* and *B. pseudocatenulatum*, optimum culture success rates in BHI were considerably lower (5.7% and 2.3%, respectively) compared to BSM-MUP (65.9% and 40.9%, respectively). This could be due to the pre-adaptation of these species to BSM-MUP, and more passages might be required to allow them to thrive in BHI, which has a different nutrient composition. These observations also suggest that the antibiotic composition of BSM-MUP did not seem to affect the culture success rates of the different *Bifidobacterium* species, and we next assess its importance for selective culture.

### Successful isolation of diverse *Bifidobacterium* species from human fecal samples using a high-throughput culturomics workflow

#### BSM supplemented with mupirocin performs better in enriching *Bifidobacterium* species from human fecal samples

**Fig 4:**
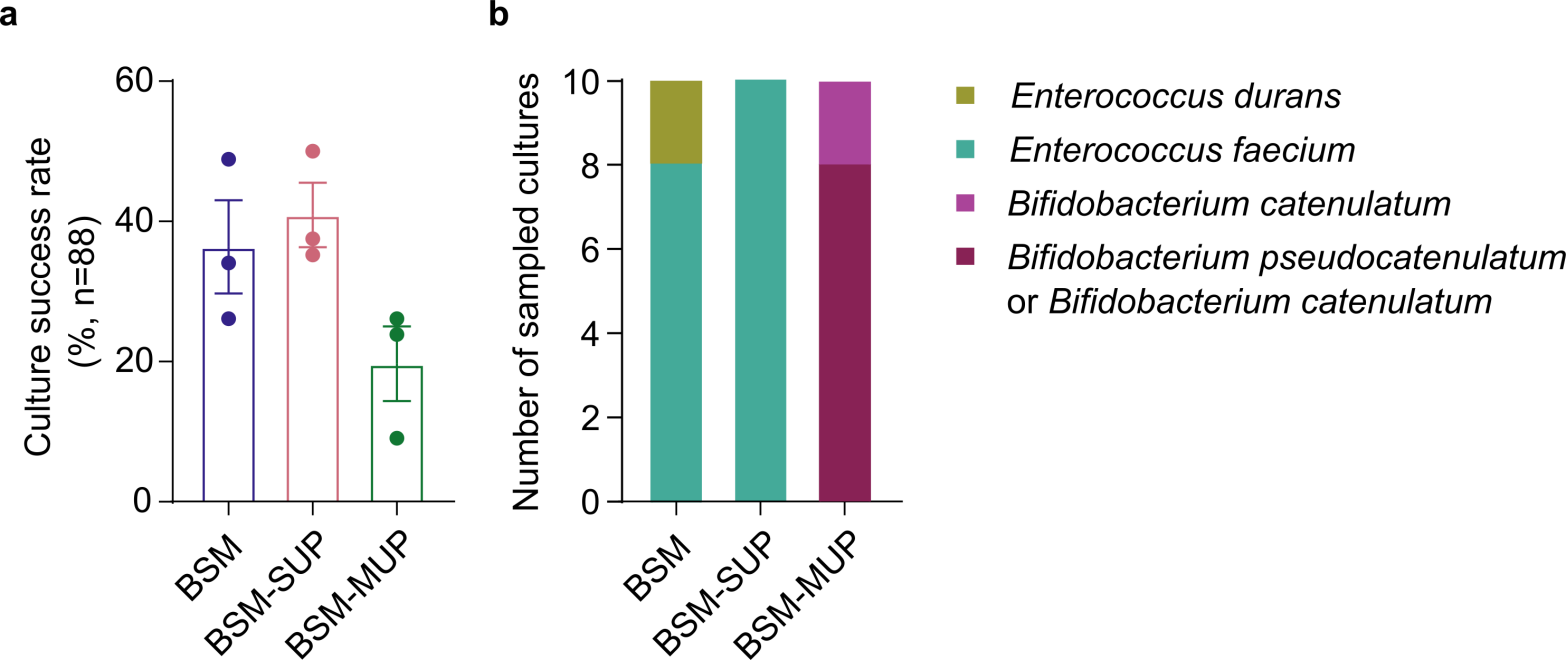
Culture success rates and identified taxa from a human fecal sample enriched in different media for 21 h. (**a**) Culture success rates, measured as the percentage of viable cultures per 96-well plate (*n* = 88), upon dispensing a 1:10^4^ stool sample enriched in BSM, BSM-SUP and BSM-MUP for 21 h. (**b**) Identity of sampled viable cultures in each medium, as determined by Sanger sequencing of the V1–V9 16S rRNA amplicon. The closest strain-level match was determined using NCBI blastn, and species-level classification was reported. Eight out of ten cultures sampled from the BSM-MUP plate had identical similarity to two different *Bifidobacterium* species.

Before proceeding to dispense multiple fecal samples, we first assessed whether BSM-MUP, despite its ability to grow pure cultures of *Bifidobacterium* species (Fig. 3a), is able to effectively enrich and select for *Bifidobacterium* species in a fecal sample compared to alternatives. We compared BSM-MUP with the base medium only (BSM) and the base medium augmented with the manufacturer’s supplement (BSM-SUP). The BSM supplement is a mixture of three different antibiotics, including polymyxin B, that inhibits a range of non-*Bifidobacterium* bacterial genera such as *Bacillus*, *Escherichia*, *Klebsiella*, *Enterobacter,* and *Pseudomonas*. A human stool sample was filtered and diluted 10^4^ times in each medium and incubated for 21 h (37°C, anaerobic conditions). Each culture was then dispensed separately into each of the media. Based on cultures after a 72 h post-dispensing incubation, BSM and BSM-SUP showed similar culture success rates (36.4% and 40.9%, respectively), while BSM-MUP yielded a relatively lower rate (19.7%; Fig. 4a). To verify that the selective media enriched specifically for *Bifidobacterium* species, a random sample of cultures (*n* = 10) from a single plate for each medium was subjected to Sanger sequencing of the V1–V9 16S rRNA amplicon. Despite showing higher culture success rates, the BSM and BSM-SUP amplicons were all from the *Enterococcus* genus (Fig. 4b; Table S2). In contrast, BSM-MUP amplicons all belonged to the *Bifidobacterium* genus, highlighting that BSM-MUP is a suitable medium to enrich for *Bifidobacterium* species from fecal samples prior to dispensing.

#### Moderate to high culture success rates are feasible for *Bifidobacterium* isolation from human fecal samples

**Fig 5:**
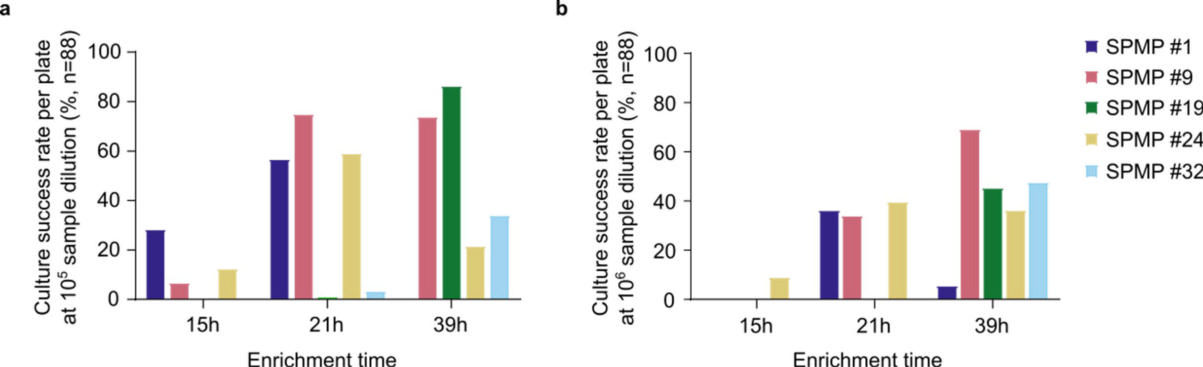
Culture success rates of five human fecal samples enriched in BSM-MUP at different dilutions and incubation time points. Culture success rates, measured as the percentage of viable cultures per 96-well plate (*n* = 88), upon dispensing five SPMP samples enriched in BSM-MUP at (**a**) 1:10^5^ and (**b**) 1:10^6^ dilution for 15 h, 21 h, and 39 h.

Moving forward with BSM-MUP as the chosen enrichment medium, we attempted to obtain isolates from five fecal samples from the *Singapore Platinum Metagenomes Project (SPMP)* cohort (36). Based on our prior observation that different *Bifidobacterium* species show variable growth rates, we performed two different dilutions of the fecal samples (10^5^ and 10^6^), and, for each dilution, enrichment in BSM-MUP was conducted for three different incubation periods (15 h, 21 h, and 39 h) before dispensing. In total, 622 cultures were obtained across SPMP samples, enrichment times, and dilutions (Fig. 5; Table S3). After 15 h of enrichment and dispensing, only a small proportion of wells had cultures for samples SPMP #1 (10^5^, 28.4%), SPMP #9 (10^5^, 6.8%) and SPMP #24 (10^5^, 12.5%; 10^6^, 9.1%), suggesting that this time point might have been too early for *Bifidobacterium* species to be enriched. Interestingly, enrichment at 21 h yielded the highest culture success rate for samples SPMP #1 (10^5^, 56.8%), SPMP #9 (10^5^, 75.0%) and SPMP #24 (10^5^, 59.1%), while 39 h enrichment recovered the most cultures from samples SPMP #19 (10^5^, 86.4%) and SPMP #32 (10^6^, 47.7%). This variation in the culture success rate as a function of the enrichment time may be due to differences in *Bifidobacterium* profiles for each enriched sample, as explored subsequently via whole-genome sequence analysis. The identity of 151 randomly sampled cultures was verified using polymerase chain reaction (PCR) amplification of the *xfp* gene, a gene exclusively found in *Bifidobacterium* species (Fig. S4). The vast majority of cultures (149/151 = 98.7%) showed a PCR product at the expected 235-bp band for *xfp*, suggesting that they contained members of the *Bifidobacterium* genus.

#### Recovery of diverse and novel *Bifidobacterium* strains in the human gut microbiome

**Fig 6:**
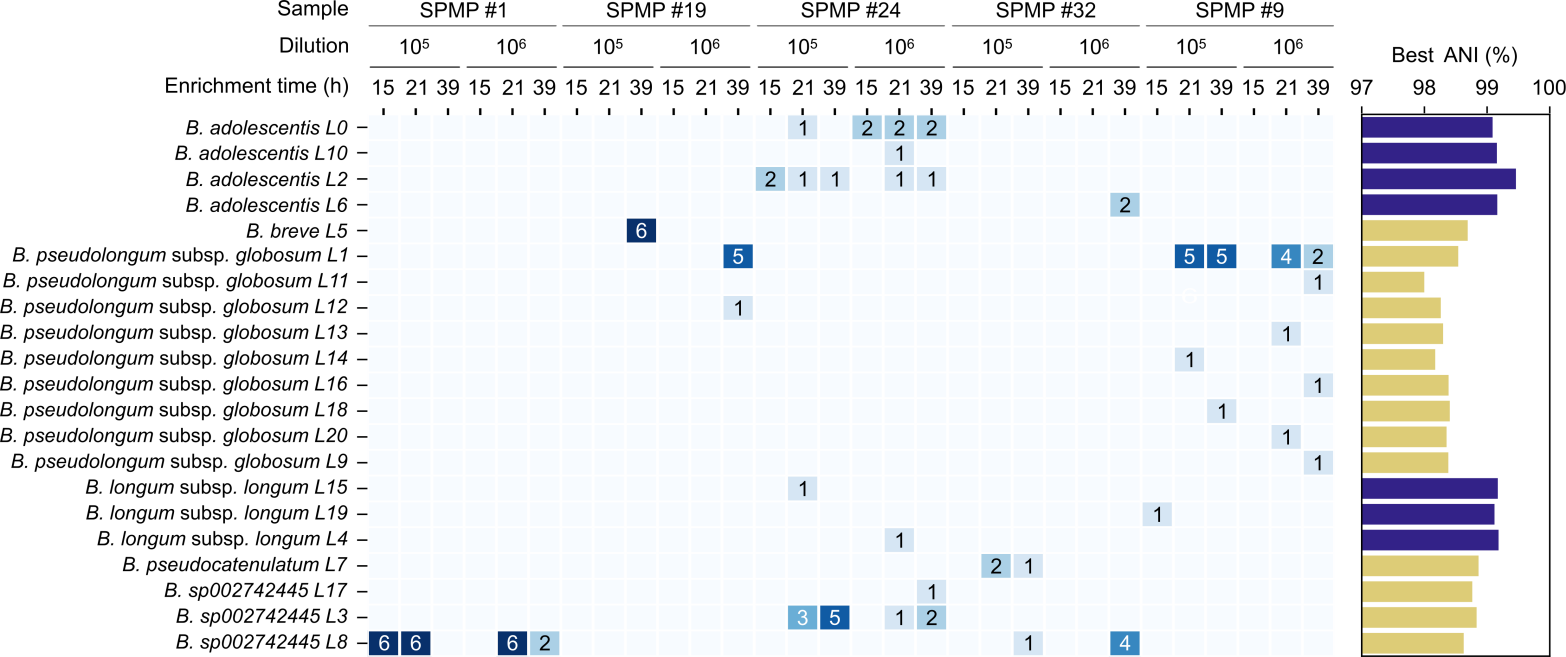
Gut *Bifidobacterium* lineages obtained using a high-throughput culturomics workflow. (Left) Lineage-level (ANI = 99.9%) representation of genomes grouped by sample ID, dilution factor, and enrichment time. Each row represents a lineage, and each column represents a dispensing run (sample/dilution/enrichment time). Heatmap annotations display the number of cultures/genomes obtained. (Right) Barplot shows the best ANI obtained for each lineage against all available *Bifidobacterium* genomes within the GTDB R220 database. *Blue*: ANI > 99.0%. *Yellow*: ANI < 99.0%.

DNA extraction, whole-genome sequencing, and genome assembly were performed for 96 out of the 622 cultures. For plates exhibiting >20% recovery rate, six wells were selected, while two wells were selected for other plates. All 96 cultures were barcoded individually and sequenced on a single Oxford Nanopore MinION flow cell. For each culture, genomes were assembled and classified at the species level against reference genomes in the GTDB R220 database (37). Out of the genomes assembled, 2/96 were assessed to have high contamination and/or low completeness values, indicating that they may not be from pure cultures and were removed from downstream analyses. Across the remaining cultures, a total of six *Bifidobacterium* species and subspecies were found, including *Bifidobacterium sp002742445* (*n* = 37), *B. pseudolongum* subsp*. globosum* (*n* = 29), *B. adolescentis* (*n* = 16), *B. breve* (*n* = 6), *B. longum* subsp. *longum* (*n* = 3), and *B. pseudocatenulatum* (*n* = 3). Circular contigs, indicating genome completeness, and an L90 value of 1 were obtained for 75.5% (71/94) and 79.8% (75/94) of the assemblies, respectively. Leveraging the high quality of assembled genomes, we clustered them using genome-wide average nucleotide identity (ANI; Fig. S5), identifying 11 unique strains (ANI = 99%) and 21 unique lineages (ANI = 99.9%), among which the majority (*n* = 14) have an ANI of less than 99% relative to publicly available genomes in GTDB (Fig. 6). This observation emphasizes the significant uncharacterized genetic diversity of *Bifidobacterium* strains within Asian gut microbiomes, which aligns with findings from our previous study using the same cohort (36). Moreover, a total of eight unique lineages of *B. pseudolongum* subsp*. globosum* (*B. globosum*) were identified based on different enrichment times (21 h and 39 h) and dilutions in sample SPMP #9 alone. Similarly, three distinct *B. adolescentis* lineages were isolated from sample SPMP #24 across culturing conditions, highlighting the ability to capture intra-subject genetic diversity with this approach. Different species also appeared at varying sample dilutions. At 39 h, only *B. breve* was isolated for the sample with 10^5^ dilution, while the same enrichment time yielded only *B. globosum* for the sample with 10^6^ dilution, underlining the importance of culturing parameters such as enrichment time and dilution factor in this workflow.

Besides the genetic diversity highlighted by the 14 lineages with an ANI of less than 99% compared to public databases, we wanted to determine whether the genomes obtained matched our previously obtained metagenome-assembled genomes (MAGs) from these samples (36) or publicly available whole-genome sequences for *Bifidobacterium* isolates (37). Interestingly, 6 out of 7 of the *B. adolescentis* and *B. longum* subsp. *longum* genomes represent novel lineages (ANI < 99.9%) when compared to only isolate-derived genomes in the GTDB database (Fig. S6). Moreover, 20 out of 21 lineages (>95%) and 10 out of 11 unique strain-level groups (>90%) that were cultured and sequenced here had no strain-level representation in GTDB isolate-derived genomes, highlighting the ability to readily expand on existing isolate genome diversity. We next compared our culture-based genomes (CBGs) with *Bifidobacterium* MAGs produced from the same stool samples (36). Out of the 14 possible pairs for species found in a CBG and MAG from the same sample, eight lineages were exclusively found within CBGs, three were found in both CBGs and MAGs, and three were found only within MAGs. As expected, the species with lineages recovered in both MAGs and CBGs showed a higher relative abundance compared to other categories, but no notable differences were observed between the CBG- and MAG-only groups (Fig. S7). Together, these results showcase the value of this high-throughput culturomics workflow for obtaining an enhanced view of *Bifidobacterium* genomic diversity within human gut microbiomes.

## DISCUSSION

### Performance characteristics of a commercially available single-cell dispenser

This study started by characterizing some of the key capabilities of a high-throughput microbial single-cell dispensing system as these can significantly impact the utility of a workflow based on it. To determine the dispenser’s doublet-generation frequency, a dual fluorescence detection method was adopted by dispensing a mixture of two fluorescent *K. pneumoniae* strains, resulting in a doublet frequency estimate of 11.5% (Fig. 1b). The doublet frequency observed using this method was higher than by visual inspection of the nozzle images post-dispensing, suggesting that the latter approach could underestimate the true number of doublets. This observation is likely due to the low resolution of the camera and the presence of dark edges in the cartridge, which can obscure cells in the *region of interest* (ROI; Fig. S8), as well as the possibility that overlapping cell images are recognized as a single cell. Other detection methods were also tested, including observation of double colonies when cells were dispensed onto agar-filled wells and identification via growth curves, but neither of these was useful in estimating the doublet frequency. Double colonies were indistinguishable from single colonies on agar (Fig. S9a). Growth curves of wells containing doublets, as identified by fluorescence detection, were also often indistinguishable from other wells (Fig. S9b). A different approach was previously described where GFP-transformed *E. coli* cells were dispensed onto glass slides and counted using a fluorescence microscope (27). This method estimated a doublet frequency of 4.0±0.4%, which is lower than the frequency observed in this study. This lower frequency could be explained by the fact that cells may be dispensed into the same spot, leading to the possibility of multiple cells contributing to the same fluorescent spot on the glass slide. The dual fluorescence detection method used in this study removes such biases from affecting the doublet frequency estimate. Through this study, we also show that the cartridge could be reused by washing three times or more with PBS, resulting in relatively low contamination rates for the next sample (Fig. S1). In addition, each PBS wash only took approximately 3 minutes to complete, making it relatively time-efficient. However, the upper limit of how many times the cartridge can be washed with PBS remains to be further tested as leakage was observed after several washes in some cases.

We have also shown in this study that the single-cell dispensing system used could preserve the global diversity of a mixed *Bifidobacterium* community. Surprisingly, even though the starting mock community was constructed based on OD_600_-colony-forming unit (CFU) calculations to pool roughly equal CFUs/species together, it was observed that the proportion of each species was unequal. Since the Sylph taxonomic profiler produces genome-length normalized taxonomic abundance values, it is likely that technical errors occurred during the construction of the mock community. In fact, the OD_600_ was measured after 6 h of incubation with its corresponding CFU/mL recorded. These species were then sub-cultured again on the subsequent day for the mock community construction. Hence, the actual cell count for these subcultures could have been slightly different from their initial cultures, although the protocol, volume of initial inoculum, and time point of harvesting were strictly followed. Nonetheless, despite these pre-dispensing disparities in the relative abundance, all nine species were obtained at a frequency >1% in all triplicates, indicating the suitability of this high-throughput culturomics approach for *Bifidobacterium* isolation (Fig. 2b). The dispensing system does not, however, appear to be free of biases with respect to different *Bifidobacterium* species, which could be due to features of its cell detection algorithm or alternatively due to biases in cell-cell interactions and clump formation. For instance, as observed under a microscope, two of the four species that were under-represented in the post-dispensing cultures (Fig. 2b), *B. animalis* and *B. bifidum*, have a more elongated shape compared to the rest of the species (Fig. S3). To test this potential bias further, experiments that vary the cell-detection parameters of the platform (such as roundness) could be explored. In addition, clumping was observed for *B. angulatum*, which might have resulted in its reduction in prevalence after dispensing. Other sources of bias, such as cell size, could be further tested to characterize the capabilities and limitations of this system.

### High-throughput single-cell isolation of *Bifidobacterium* species

Having shown that the single-cell dispenser is capable of isolating diverse *Bifidobacterium* species, we aimed to examine different culturing factors that could affect its recovery rates. By dispensing pure cultures of the different *Bifidobacterium* species into both BSM-MUP and BHI, we were able to compare the optimum culture success rates between the two different post-dispensing media. We observed that for most of the species, the culture success rates were similar between BSM-MUP and BHI, indicating that post-dispensing cultures could survive in both media, even with the antibiotic pressure posed by BSM-MUP (Fig. 3). Additionally, for two of the species, *B. animalis* and *B. pseudocatenulatum*, recovery was better in BSM-MUP compared to BHI, which could be due to their adaptation to the BSM-MUP environment in the pre-dispensing culture. Another possible explanation is the fact that BSM is specifically formulated to stimulate the growth of *Bifidobacterium* species (38), while BHI is a general-purpose medium.

To further develop our workflow, we also assessed the performance of BSM-MUP compared to alternatives such as BSM base medium and BSM-SUP. Despite showing a lower culture success rate compared to its alternatives (Fig. 4a), BSM-MUP is the only medium that successfully yielded *Bifidobacterium* cultures at high frequencies. When cultures were sampled at random from each medium, BSM and BSM-SUP cultures were all identified as *Enterococcus* species (Fig. 4B; Table S2). Although BSM has been reported to inhibit the growth of *Enterococcus* species, we suspect that its primary use for quality control of dairy products has meant that it has not been extensively tested with *Enterococcus* species from the human gut (e.g., *E. faecium*). On the other hand, MUP is a selective agent that has been shown to be effective against *E. faecium* and *E. faecalis* but non-inhibitory to *Bifidobacterium* (39). We also further examined the efficacy of MUP by culturing *E. faecium* and *Bifidobacterium* species isolates in BSM-MUP. The results show complete inhibition of *E. faecium* and growth of *Bifidobacterium* species after 48 h and align with the lack of *Enterococcus* and dominance of *Bifidobacterium* among BSM-MUP cultures (Fig. S10). In this study, we did not compare our results with trans-galactosylated oligosaccharide propionate, supplemented with mupirocin (TOS-MUP), that is also designed for *Bifidobacterium* isolation, but with lower reported specificity compared to BSM-MUP (38). Furthermore, compared to TOS-MUP, which is only available as agar, BSM-MUP allows for enrichment in liquid cultures. Liquid cultures remove the need for additional steps of streaking and colony resuspension before dispensing, which makes them more compatible for use with the single-cell dispenser.

Other factors, such as tolerance to brief oxygen exposure, could also limit recovery success upon dispensing. For instance, *B. adolescentis* achieved a maximum culture success rate of 10.2% in BSM-MUP (Fig. 3a), which is lower than the rest of the species and could be due to the reported heightened sensitivity to aerobic conditions in this species (40). In our present study, the dispenser was set up in a rigid anaerobic chamber, which is prone to minor leakage of oxygen through its walls. The dispensed 96-well plates were then transferred to a vinyl anaerobic chamber for incubation. While we used anaerobic gas-generating sachets in a sealed zip-lock bag during the transfer of the samples between the chambers, there is still the risk of low levels of oxygen exposure that could have impacted the recovery of *B. adolescentis*.

Varying the incubation period pre-dispensing seemed to affect the culture rates of individual *Bifidobacterium* species (Fig. 3a). Incubation periods of 6 h and 10 h produced the highest culture success rates in BSM-MUP for eight of the species, while *B. breve* was best isolated at 26 h. These time points correspond to different growth phases for these species (Fig. 2a), which suggests that each species may have its unique growth phase preference for enabling recovery post-dispensing. Thus, we determined that varying the incubation period is essential for obtaining better culture success rates and diversity from a mixed community. Similar findings were observed when we isolated *Bifidobacterium* species from stool samples subsequently. For example, the different enrichment periods (15 h, 21 h, and 39 h) yielded different species profiles in the 10^5^ dilution experiment for SPMP sample #24 (Fig. 6).

### *Bifidobacterium* culture profiles from human fecal samples

When the SPMP samples were enriched in BSM-MUP and dispensed, high recovery rates were observed at different time points, depending on the sample and dilution factor. Peak culture success rates were observed for SPMP #1, #9, and #24 at 21 h (10^5^ dilution) and for SPMP #19 (10^5^ dilution) and SPMP #32 (10^6^ dilution) at 39 h (Fig. 6). This was possibly due to the unique *Bifidobacterium* species profiles of each sample, giving rise to different growth rates. Out of the 151 cultures tested across SPMP samples, dilutions, and time points, 149 (98.7%) were shown to be positive for the *Bifidobacterium* marker gene *xfp* (Fig. S4). The dilution factor when preparing each sample also likely played a role in recovering different species of *Bifidobacterium*. By comparing the 10^5^ and 10^6^ dilutions for sample SPMP #19, it was observed that the two dilutions yielded completely different species (Fig. 6). Nevertheless, this dispensing workflow was able to achieve highly specific isolation of *Bifidobacterium* species that can be leveraged for further large-scale isolation studies, while future studies could experiment with other dilution factors and enrichment periods to identify optimal conditions in a sample and species-specific manner.

Among the 96 SPMP cultures sequenced, whole-genome analyses revealed a variety of different *Bifidobacterium* species present across samples, including *B. adolescentis*, *B. breve*, *B. longum* subsp*. longum*, *B. pseudocatenulatum*, *B. pseudolongum* subsp*. globosum,* and *Bifidobacterium sp002742445. B. pseudolongum* subsp*. globosum (B. globosum)* is often considered a subspecies of *B. pseudolongum*, a species typically found in the bovine rumen (41), even though *B. globosum* has been previously reported in the human gut in a Chinese cohort (42). Some studies have suggested a role for *B. pseudolongum* in reducing triglyceride levels and suppressing non-alcoholic fatty liver disease (NAFLD)-related complications through investigations in murine models (43, 44). Thus, the isolation of *B. globosum* in the SPMP cohort provides an avenue for further investigation into the role of this species in human health. *Bifidobacterium sp002742445* was a candidate species identified in the human gut with high similarities to *B. catenulatum* and *B. kashiwanohense* (45) and recently was formally named *B. hominis* (30). Despite the recency of its discovery, some studies have suggested beneficial characteristics for this candidate species based on the genomic analysis. For instance, *Bifidobacterium sp002742445* was previously found to be the gut bacterial species that has the second highest percentage of polyphenol utilization genes within the Unified Human Gastrointestinal Genome database, which suggests that it may play an important role in polyphenol metabolism in the human gut (46). Its frequent isolation from fecal samples in Singapore provides an opportunity to further characterize the functional roles of this species and its potential impact on human health.

Across the genomes assembled, a total of 21 lineage-level clusters were identified at 0.1% divergence (Fig. 6). While only a single lineage was isolated for some species (and subjects) such as *B. pseudocatenulatum* and *B. breve*, four unique lineages were observed for *B. adolescentis*, with three of the four clusters originating from the same sample (SPMP #24), highlighting intra-subject diversity. Inter-subject diversity was also observed as three unique lineages were identified for *B. longum* subsp*. longum* and *Bifidobacterium sp002742445* across four samples (SPMP #1, SPMP #9, SPMP #24, and SPMP #32). In contrast, *B. globosum* found in both SPMP #9 and SPMP #19 also belonged to the same lineage (*B. globosum* L1). As such, both inter-sample similarity and diversity were observed in the genomes obtained. Additionally, among the 21 lineages detected, 14 showed low similarity to publicly available genomes in the GTDB database, including 20 with no genomes from isolates (ANI < 99%, Fig. 6). These potentially novel lineages were found across four different species, including *B. breve*, *B. globosum*, *B. pseudocatenulatum,* and *Bifidobacterium sp002742445*. Further phenotypic and genomic characterization could help define distinct functional features of these genetically diverged lineages. Taken together, these findings highlight the utility of a single-cell dispenser-based workflow to rapidly capture both species and strain-level *Bifidobacterium* diversity in fecal samples.

### Limitations and challenges

While the imaging and selection capabilities of the single-cell dispenser accelerate the culturomics workflow, it is not the only factor contributing to culturing success. The choice of culture conditions plays a critical role in the outcome. Without prior enrichment, dispensing raw samples may result in low yields due to the presence of a significant proportion of dead bacterial cells or stool debris (24). Furthermore, even when cells remain viable, inappropriate media or culturing conditions (e.g., insufficient time for enrichment and improper oxygen availability) can still hinder culture success. While optimization of these parameters follows similar principles to traditional culturing methods, the single-cell dispensing platform offers the advantage of increased speed and throughput in the experimental workflow. However, using general media such as Luria Bertani (LB) or BHI for enrichment can lead to the dominance of fast-growing species, skewing the microbial profile and limiting the recovery of slower-growing organisms. Therefore, careful consideration of alternative media and culturing conditions is essential to capture a better representation of the microbiome, though some bias is inevitable following enrichment. To expand this workflow for use with other targeted species, selective media or antibiotics may be necessary. Specialized enrichment procedures should be formulated for fastidious bacteria as well by supplementing the media with specific nutrients and growth factors.

Additionally, the pretreatment of samples will necessarily vary based on the sample type. Wastewater samples, for example, require concentration due to their inherent dilution, which may delay single-cell dispensing. Soil samples, on the other hand, may require careful bacterial separation to avoid clogging of the dispensing cartridge by coarse particles. Fine soil particles can be similar in size to bacteria and may be dispensed alongside viable cells, lowering recovery rates. One of the downsides of some single-cell dispensers can be their inability to distinguish between living cells and nonliving particles, which can impact their overall yield. While the system that we experimented with here has demonstrated utility with *Bifidobacterium* sp., further validation and optimization will be needed to extend its applicability to other microbial groups and sample types, enabling broader applicability across diverse species.

### Conclusion

Our study has highlighted the utility of a single-cell dispensing-based workflow to accelerate strain isolation for an important gut bacterial genus with applications in probiotic development. The performance of the single-cell dispenser was first evaluated, revealing a high single-cell dispensing frequency. In addition, BSM-MUP was demonstrated to effectively enrich fecal samples for capturing *Bifidobacterium* species diversity, with the enrichment duration playing a key role in successful recovery of various species. Building on these findings, we successfully obtained 622 cultures from fecal samples with a high-throughput workflow, the vast majority of which represent *Bifidobacterium* species. These cultures provide an important resource to study the functional properties of poorly characterized species such as *B. globosum* and *Bifidobacterium sp002742445* as well as the novel lineages highlighted in this study. While case-specific modifications will likely be required for the isolation of other organisms, we hope that the protocol established here can serve as a template that can be leveraged for the high-throughput isolation of other gut bacterial genera as well, paving the way for more in-depth characterization of functional diversity within gut microbiomes through high-throughput culturomics.

## MATERIALS AND METHODS

### Bacterial strains and growth conditions

Two strains of *Klebsiella pneumoniae* SGH10 were obtained as a gift from Prof. Gan’s lab (National University of Singapore) to be used for determining the doublet dispensing frequency using a single-cell dispensing system. Both *K. pneumoniae* strains were transformed with pKPC2 (GenBank, MN542377), each with different fluorescence genes (sfGFP1 or mApple). These strains were cultured separately in LB broth with 50 µg/mL kanamycin at 37°C for 72 h under aerobic conditions. In addition, a total of nine bacterial strains of *Bifidobacterium* species from DSMZ were also used: *B. catenulatum* subsp*. catenulatum* B669 (DSM 16992), *B. adolescentis* E194a (Variant a; DSM 20083), *B. angulatum* B677 (DSM 20098), *B. pseudolongum* PNC-2-9G (Biotype a; DSM 20099), *B. animalis* subsp*. animalis* R101-8 (DSM 20104), *B. breve* S1 (DSM 20213), *B. longum* subsp*. infantis* S12 (DSM 20088), *B. pseudocatenulatum* B1279 (DSM 20438), and *B. bifidum* Ti (DSM 20456). All *Bifidobacterium* strains were maintained on BSM-MUP (Millipore, Darmstadt, Germany) with 0.01% L-cysteine (Sigma-Aldrich, Darmstadt, Germany) and under anaerobic conditions (N_2_ [75%], CO_2_ [20%], and H_2_ [5%]) at 37°C. The composition of each media is listed in File S1.

### Growth curves and OD_600_-CFU relationships

To assess the doublet dispensing frequency of the single-cell dispenser, the two fluorescent *K. pneumoniae* SGH10 strains were diluted in LB broth to OD_600_ = 0.2 and incubated at 37°C for 9 h. At every 1 h interval, OD_600_ was measured to construct a growth curve for each culture, which was then serially diluted (10^−4^–10^−8^) and plated in triplicate spots of 5 µL on LB agar for CFU counting. The OD_600_-CFU relationship for each strain was plotted (Fig. S11). Approximately 10^7^ CFU/mL of each fluorescent strain was combined for single-cell dispensing. To assess the single-cell sorter’s dispensing biases, a mock community consisting of the nine *Bifidobacterium* species was used. The OD_600_-CFU relationship of each *Bifidobacterium* species was measured according to the protocol above with slight modification. Briefly, the overnight culture of each species was diluted individually in BSM-MUP broth to OD_600_ = 0.1 and incubated at 37°C for 16 h under anaerobic conditions. OD_600_ was then measured at 2 h intervals for each species to construct growth curves (Fig. 1c). After performing CFU counting for each species at the 6 h time point, each species was then re-cultured separately in BSM-MUP broth at 37°C for 6 h, and 3.57 × 10^8^ CFU/mL of each culture was combined to form the mock community for dispensing.

### Single-cell dispensing

A single-cell dispensing system (B.SIGHT v1, Cytena GmBH, Germany) was installed within an anaerobic chamber. Samples were kept in a zip-lock bag containing an AnaeroGen 2.5L sachet (Oxoid, UK) when being transferred between the chambers to reduce the risk of oxygen exposure. All samples were filtered using a 20 µm pluriStrainer (pluriSelect Life Science, Germany) before being loaded into the cartridge using nonfiltered sterile 200 µL pipette tips. The cell density of each sample was estimated using the number of cells detected per minute by the camera installed in the machine. Samples with excessively high cell density were diluted with pre-reduced PBS. An optimal density of ~200 to 500 cells/min was used for all fecal samples. The default settings for repetitions per minute during agitation were configured to 9 for a 20 µL volume, while the idle shots per minute for dispensing were set to 60. For the tracking algorithm, a detection radius (ROI) of 300, a detection threshold (ROI) of 3, and an FI detection threshold (ROI) of 4 were utilized. In the automation settings, the server port number, dispensing event warning threshold, and dispensing event error threshold were all set to 3,000. For droplet quality control during dispensing, the following parameters were applied: a vertical delay of 3.5, a horizontal delay of 0.0, a stroke length of 3 µm, and a stroke velocity of 75 µm/ms for all dispensing events. Cell roundness and size parameters were also set to 0–1 and 0–10 µm, respectively, for all dispenses. Single cells were dispensed into the first 11 columns (A1–H11) of a Corning round-bottom 96-well plate containing 200 µL of liquid media per well. The last column of each plate was used for blanks (wells A12–C12), nontemplate controls (NTCs; wells D12–G12), and a 400-droplet positive control (well H12). NTC droplets were detected by the camera to be empty and served as a negative control for all dispenses. After dispensing, the 96-well plates were incubated at 37°C. Each nozzle image was carefully examined to determine whether a second cell was present within the ROI during the dispensing process.

### Post-dispensing cartridge washing

Upon dispensing, the sample was removed from the cartridge and replaced with 70 µL of PBS. The remaining cells inside the cartridge were discharged for ~3 min by choosing the “Collect samples” function in the x.sight software (version 1.4.1; Cytena GmBH, Germany). The number of leftover cells was detected by the ROI of the live image on the camera and plotted on the “Results” graph. After the 3-minute discharge, the PBS was replaced, and the cartridge was washed for another 2–4 times until no cell could be detected. The contamination rate was estimated after each washing step by taking the ratio of leftover cells detected per minute to the original cell density detected per minute.

### Fluorescence detection

Six 96-well Corning plates containing fluorescent *K. pneumoniae* dispensed were incubated in aerobic conditions at 37°C for 24 h. Fluorescence from individual wells was detected and quantified using a Tecan Spark Multimode plate reader. For sfGFP1 fluorescence, excitation and emission wavelengths were set at 475 nm and 515 nm, respectively. For mApple fluorescence, excitation and emission wavelengths were set at 560 nm and 600 nm, respectively. Fluorescence measurements were normalized by the mean background fluorescence of NTC wells, followed by a min-max standardization to account for different fluorescence intensities between sfGFP1 and mApple. Positive fluorescence signal thresholds were defined as two standard deviations from the mean background fluorescence.

### *Bifidobacterium* cell culture dispensing

All nine *Bifidobacterium* isolates mentioned above were individually diluted to OD_600_ = 0.1 in BSM-MUP broth. After 6 h, 10 h, and 26 h of incubation, the cell cultures were diluted to 10^−2^ in PBS and dispensed into four 96-well plates, with two plates containing BSM-MUP and another two plates containing BHI media (Oxoid, UK). All media and PBS were pre-equilibrated in the anaerobic chamber for at least 48 h before dispensing. After 48–72 h of incubation, the number of wells with growth was counted and recorded. Cellular morphology of each species was imaged with a light microscope (BX23, Olympus).

### Stool sample collection

Five independent human fecal samples were obtained from the SPMP, labeled (in order) SPMP #1, SPMP #9, SPMP #19, SPMP #24, and SPMP #36. Informed consent was obtained from each subject, and all protocols for this study were approved by the National University of Singapore Institutional Review Board (IRB reference number H-17-026). All SPMP samples were collected from healthy subjects using a BioCollector kit (BioCollective, Colorado, USA). Samples were double-bagged and kept in a −20°C polystyrene box. Samples were stored in the anaerobic chamber (N_2_ [75%], CO_2_ [20%], and H_2_ [5%]) before being homogenized, sealed, and frozen at −80°C for long-term storage.

### Enrichment culture with stool samples and dispensing

Each stool sample (100 mg) was transferred to a 1.5 mL Eppendorf tube and homogenized by pipetting in 1 mL of sterile PBS, which had been pre-equilibrated for 48 h in the anaerobic chamber. To evaluate the efficiency of different selective media, the homogenized sample (SPMP #1) was then serially diluted in PBS to 10^−3^ and transferred to either BSM, BSM-SUP (Millipore, Darmstadt, Germany), or BSM-MUP to make a 10^−4^ dilution. After 21 h of incubation and enrichment, the cell cultures were filtered through a 20 µm cell strainer to remove large non-organic residues and serially diluted to 10^−4^ in PBS for dispensing into 96-well plates containing respective media. After 48–72 h of incubation, the identity of each species was verified using V1–V9 16S PCR and Sanger sequencing. Once suitable culturing media and conditions were determined, high-throughput isolation of *Bifidobacterium* species from all stool samples was performed using the above procedure with slight modifications. Specifically, the five SPMP stool samples enriched in BSM-MUP were diluted to 10^−5^ or 10^−6^ and enriched for 15 h, 21 h, and 39 h before dispensing into BSM-MUP.

### DNA extraction and purification

To extract DNA from the cultures in 96-well plates, MGISP-960 (MGI Tech Co., Ltd., China), a high-throughput automated sample preparation system, was adapted to automate the extraction process. Post-dispensing liquid cultures in a 96-well Corning plate were transferred to a 1.3 mL deep-well plate and centrifuged at 4,000 rpm for 15 min at room temperature and pressure. The supernatant was then discarded, leaving cell pellets of size 0.3–0.5 cm. The deep-well plate with cell pellets was then sealed and temporarily stored at 4°C. Lysozyme (20 mg/mL) was prepared in a lysis buffer consisting of 1.2% Triton X-100, 20 mM Tris-HCl pH 8.0, and 2 mM EDTA in nuclease-free water (NFW). An in-house MGISP-960 protocol was then run. Briefly, cell pellets were resuspended in a 90 µL lysozyme-containing lysis buffer and incubated at 37°C for 30 min. Both 12.5 µL Proteinase K and 100 µL AL Buffer (Qiagen, USA) were then added for chemical lysis at 56°C for 15 min. After chemical lysis, 170 µL 1× AMPure magnetic beads were added, mixed, and incubated on a shaker for 10 min at room temperature. The samples were then moved to a magnetic stand for 5 min before all the supernatants were removed. The beads were washed twice with 150 µL of 80% ethanol, and DNA was eluted in 75 µL NFW. Post-extraction quantification of the final DNA yield was performed using Qubit 4.0.

### PCRs

The components of two PCRs for the V1–V9 16S region and *xfp* gene fragment are detailed in Table S4. The V1–V9 16S rRNA PCR was used to identify species-level taxonomy of the dispensed isolates by Sanger sequencing. The *xfp* gene PCR was used to identify whether the cultures obtained belonged to the *Bifidobacterium* genus. The following primers were used:

For 16S rRNA PCR (47):

Forward primer: 5′-AGRGTTYGATYMTGGCTCAG-3′

Reverse primer: 5′-CGGYTACCTTGTTACGACTT-3′

For *xfp* PCR (48):

Forward primer: 5′-ATCTTCGGACCBGAYGAGAC-3′

Reverse primer: 5′-CGATVACGTGVACGAAGGAC-3′

The PCR protocols were performed as described in Table S5 using a thermal cycler. For the 16S rRNA PCR, PCR products were incubated with 1 × AMPure magnetic beads for 5 min and left on a magnetic stand for another 5 min. The solvent was then removed, and beads were washed twice with 80% ethanol. 16S rRNA PCR products were then eluted using an elution buffer and run on a Tapestation gDNA screentape (Agilent, USA). The expected band size was 1.5 kbp. PCR products were then sent for Sanger sequencing (1st Base, Singapore), and the closest strain-level matches were determined using blastn against the 16S ribosomal RNA (Bacteria and Archaea type strains) database (updated: 2025/07/18). Manual inspection of chromatograms from Sanger sequencing did not show any overlapping peaks suggestive of mixed cultures. For the *xfp-*PCR, PCR products were directly run on a 2% agarose gel supplemented with 1× gel red for visualization. The expected band size was 235 bp.

### Whole-genome sequencing on a nanopore system

There were two nanopore sequencing runs in the study. The first run was to compare the composition of the mock community pre- and post-dispensing to check for any substantial species-specific biases in single-cell dispensing. For this purpose, DNA samples were prepared following the SQK-NBD114.24 protocol (Oxford Nanopore, UK). Briefly, for each plate with dispensed isolates, equimolar amounts of extracted DNA from each well were pooled together to form a single 400 ng sample. The extracted DNA from the original mock communities (pre- and post-filtering) was also aliquoted as controls. For each sample, DNA repair and end-prep were performed by adding Ultra II End-prep enzyme mix and FFPE DNA repair mix as per the protocol. The reactions were incubated at 20°C for 15 min and 65°C for 5 min. Native barcode ligation was then done by incubating the samples with four native barcodes (NB01-04) and Blunt/TA Ligase Master Mix at room temperature for 20 min. EDTA (10%) was then added to terminate the reaction. DNA was then purified with AMPure XP Beads followed by two 80% ethanol washes and eluted in NFW after each step. All barcoded DNA was subsequently pooled together for adapter ligation by incubating with Native Adapter (NA) and Quick T4 DNA Ligase at room temperature for 20 min. The DNA library was then purified once more with AMPure XP Beads followed by two Long Fragment Buffer washes and elution in Elution Buffer, as per the protocol. DNA yield was determined using Qubit 4.0, and 10–20 fmol of the DNA library was loaded onto a Flongle flow cell (Oxford Nanopore, UK) primed with Flow Cell Flush, 50 mg/mL Bovine Serum Albumin, and Flow Cell Tether. Sequencing was run for 24 h. The second Nanopore sequencing run was to identify *Bifidobacterium* species represented in the cultures and to construct their genomes. DNA samples were prepared following the SQK-NBD114.96 protocol (Oxford Nanopore, UK). DNA extracted (200 ng) from each of the 96 cultures selected was repaired, end-prepped, and barcoded separately (NB01-96), as described previously. DNA was purified with AMPure XP Beads followed by two 80% ethanol washes and eluted in NFW after each step. The barcoded DNA was then pooled together to form a single sample before adapter ligation and flow cell loading. For this round of sequencing, a MinION flow cell (Oxford Nanopore, UK) was used, and sequencing was run for 72 h.

### Whole-genome analysis

For the first sequencing run, raw sequencing reads were processed and analyzed using Sylph (v.0.6.1) (49) against the GTDB R220 representative genomes database (37). For the second sequencing run, raw sequencing reads were basecalled with Dorado (v7.6.7), the latest version of the basecaller available at the point of sequencing, generating a total of 9.2 Gbp corresponding to an average of 96 Mbp per sample. For each barcode, reads were processed using an in-house whole-genome assembly pipeline, briefly described as follows. An initial *de novo* assembly was generated with Flye (v.2.9.5) (50), and potential plasmid assemblies were further improved using plassembler (v.1.6.2) (51). Whole-genome assemblies were then reoriented with Dnaapler (v.1.2.0) (52). The resulting assemblies were first screened at the species level against the GTDB R220 database with GTDB-Tk (v.2.4.0) (53) using the “classify_wf” method. Two genomes with high contamination (>90%) and/or low completeness (<50%) were identified with CheckM2 (v.1.1.0) (54) and removed from subsequent analyses. To further characterize *Bifidobacterium* genomic diversity, assemblies were compared to all *Bifidobacterium* genomes found within GTDB R220 and to each other using Mummer4 (v.4.0.1) (55) nucmer and dnadiff. A threshold of 75% and 50% of minimum genome alignment for self and GTDB comparisons, respectively, was defined to avoid spurious ANI values. Isolates were clustered using agglomerative clustering with average linkage, and thresholds of 99% ANI and 99.9% ANI were used to identify strain and lineage-level groups, respectively. Comparison with SPMP metagenomes was done based on MAGs obtained from our previous study (36). MAGs were compared against the same GTDB R220 database with Skani (v.0.2.2) (56) and assigned a species based on ANI≥95%.

## Data Availability

The sequencing data for this study are available from the European Nucleotide Archive (ENA) at https://www.ebi.ac.uk/ena/browser/view/PRJEB98585, with reference number PRJEB98585.

## SUPPLEMENTAL MATERIAL

File S1 spectrum.03033-25-s0001.pdf Composition of various media used in this study.

Supplemental figures spectrum.03033-25-s0002.pdf Figures S1 to S11.

Supplemental tables spectrum.03033-25-s0003.xlsx Tables S1 to S5.

ASM does not own the copyrights to Supplemental Material that may be linked to, or accessed through, an article. The authors have granted ASM a non-exclusive, world-wide license to publish the Supplemental Material files. Please contact the corresponding author directly for reuse.
