# Supplementary material for: High-throughput single-cell isolation of Bifidobacterium strains from the human gut microbiome
Source: Microbiol Spectr. 2025 Dec 30;14(2):e03033-25. doi: 10.1128/spectrum.03033-25 (PMC12889045; doi:10.1128/spectrum.03033-25)
Supplement: File S1 — Composition of various media used in this study. [file spectrum.03033-25-s0001.pdf]

Supplementary file 1: Composition of various media used in this study

<u>Luria Bertani (LB) broth (Miller – Novagen, Darmstadt, Germany)</u>

5g yeast extract, 10g peptone from casein, 10g sodium chloride, and 1L distilled water.

<u>Brain Heart Infusion (BHI) media (Oxoid, UK)</u>

12.5g brain infusion solids, 5.0g beef heart infusion solids, 10.0g proteose peptone, 2.0g glucose, 5.0g sodium chloride, 2.5g disodium phosphate, and 1L distilled water.

<u>Bifidus Selective Medium (BSM) Broth suitable for microbiology, NutriSelect® Basic, (Millipore, Darmstadt, Germany)</u>

42.5g BSM powder (containing peptone, meat extract, yeast extract, dextrose, sodium chloride, reducing and buffering agents, selective salts, metabolic by-products-detoxifying and glycolysis-inhibiting compounds, three antibiotics inhibiting Bacilli, Enterobacteriaceae and *Pseudomonas*, as well as azo compound) and 1L distilled water.

Whenever required, lithium mupirocin (0.05g/L) or BSM Supplement (0.116g/L; detailed formula is unavailable from manufacturer) was supplemented to BSM broth to prepare BSM-MUP or BSM-SUP respectively.

**The detailed composition of BSM broth was not provided by the manufacturer.*
