# Supplementary material for: High-throughput single-cell isolation of Bifidobacterium strains from the human gut microbiome
Source: Microbiol Spectr. 2025 Dec 30;14(2):e03033-25. doi: 10.1128/spectrum.03033-25 (PMC12889045; doi:10.1128/spectrum.03033-25)
Supplement: Supplemental figures — Figures S1 to S11. [file spectrum.03033-25-s0002.pdf]

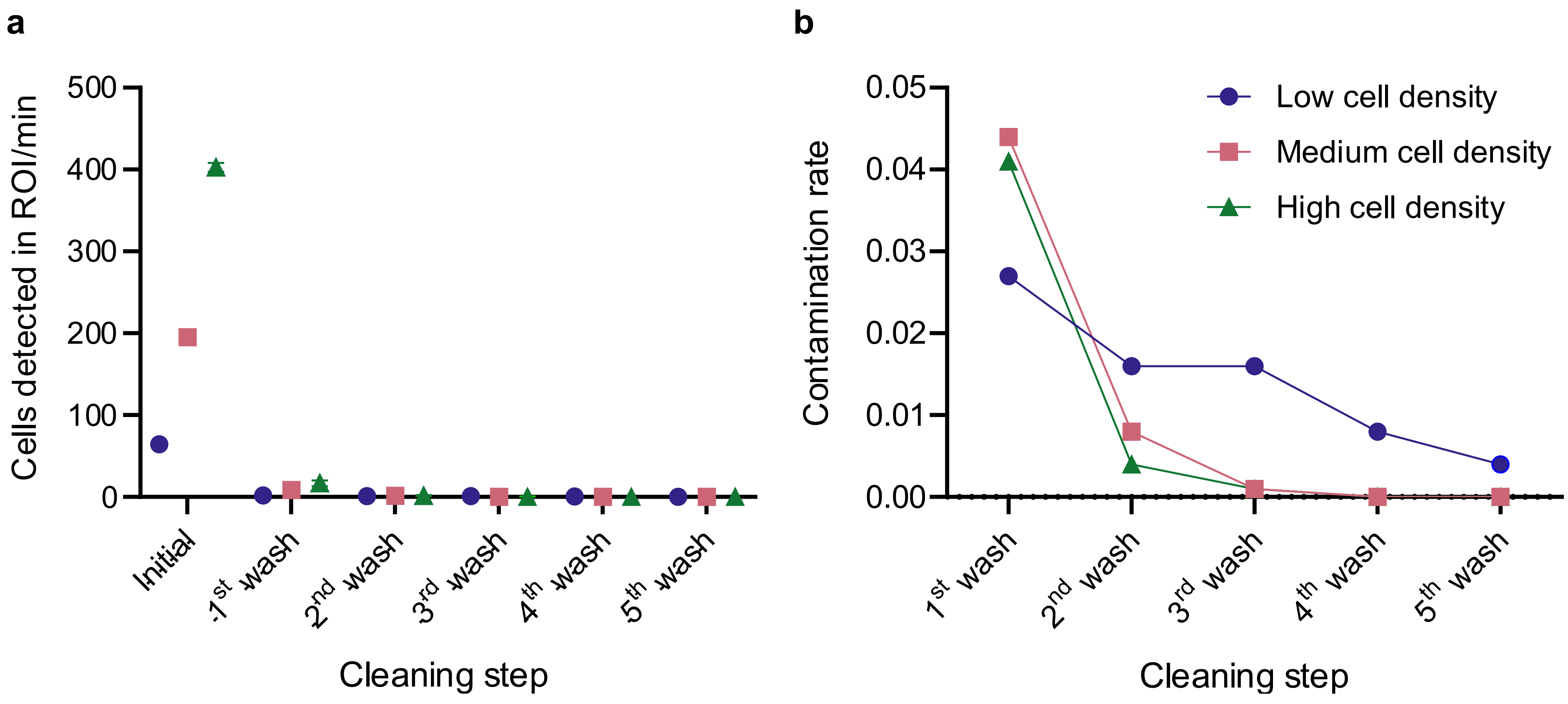

Supplementary Figure 1. Impact of wash steps on contamination across samples. (a) Graph showing the number of cells detected in the ROI of B.SIGHT's camera (per minute) after each round of PBS wash of the cartridge. (b) Plot showing contamination rate (number of cells detected per minute divided by pre-wash count) after each round of wash. Samples were diluted to low (blue, 64.4 cells/min), medium (red, 195.4 cells/min) or high cell density (green, mean 402.6 cells/min). Mean and standard error of recordings were reported based on n=3 replicates.

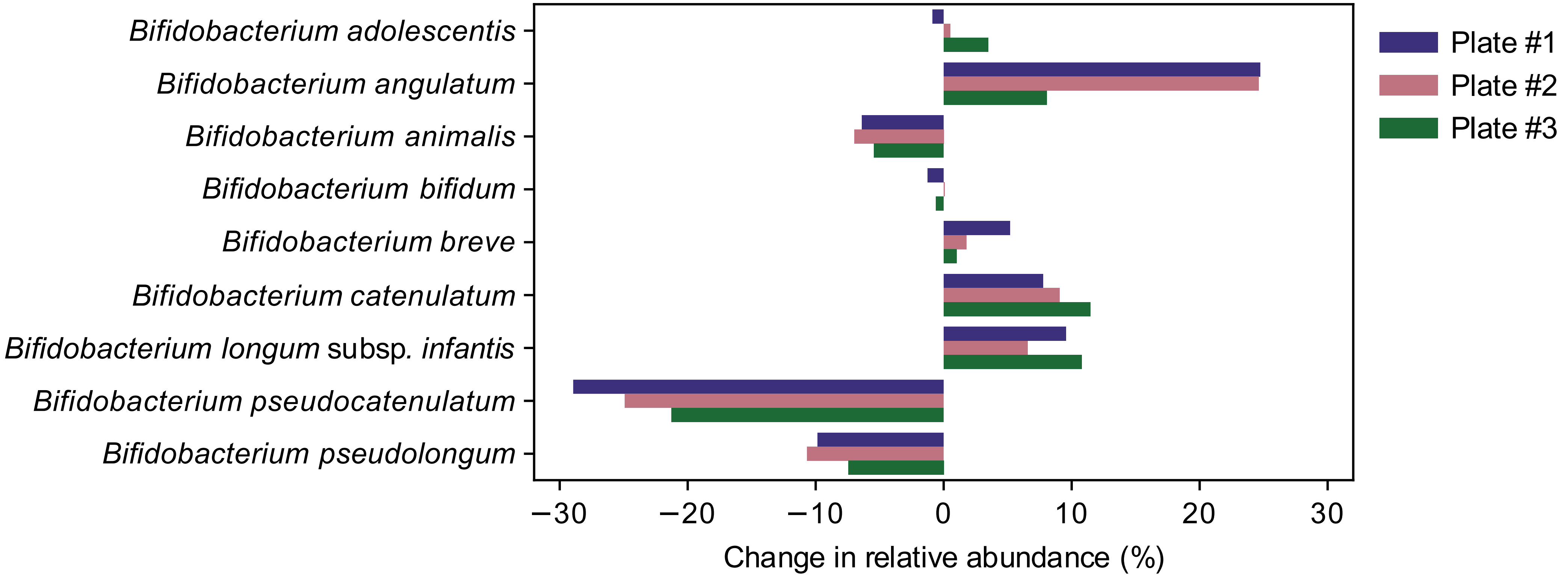

Supplementary Figure 2. Changes in relative abundances of individual *Bifidobacterium* species in the mock community after dispensing. Changes were computed relative to the filtered mock community.

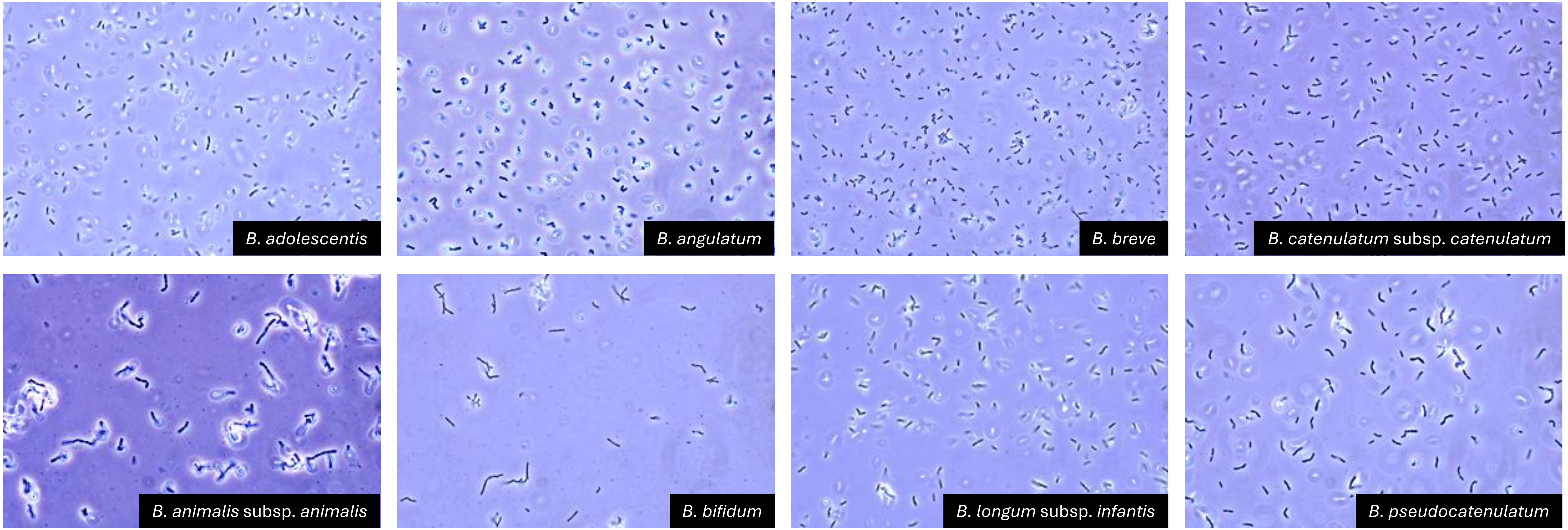

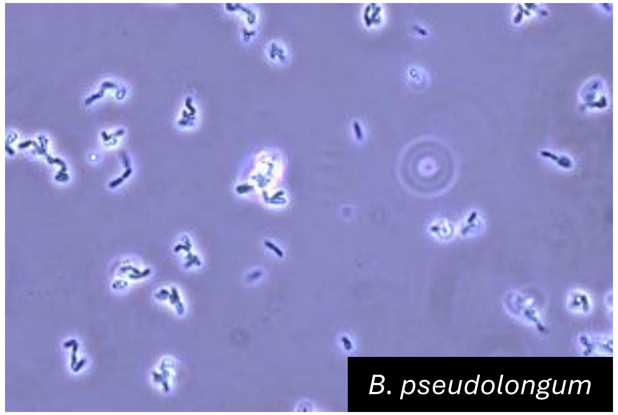

Supplementary Figure 3. Microscope images of nine *Bifidobacterium* species from the mock community. Images were taken at the mid-log phase of each species and highlight the morphological differences across species.

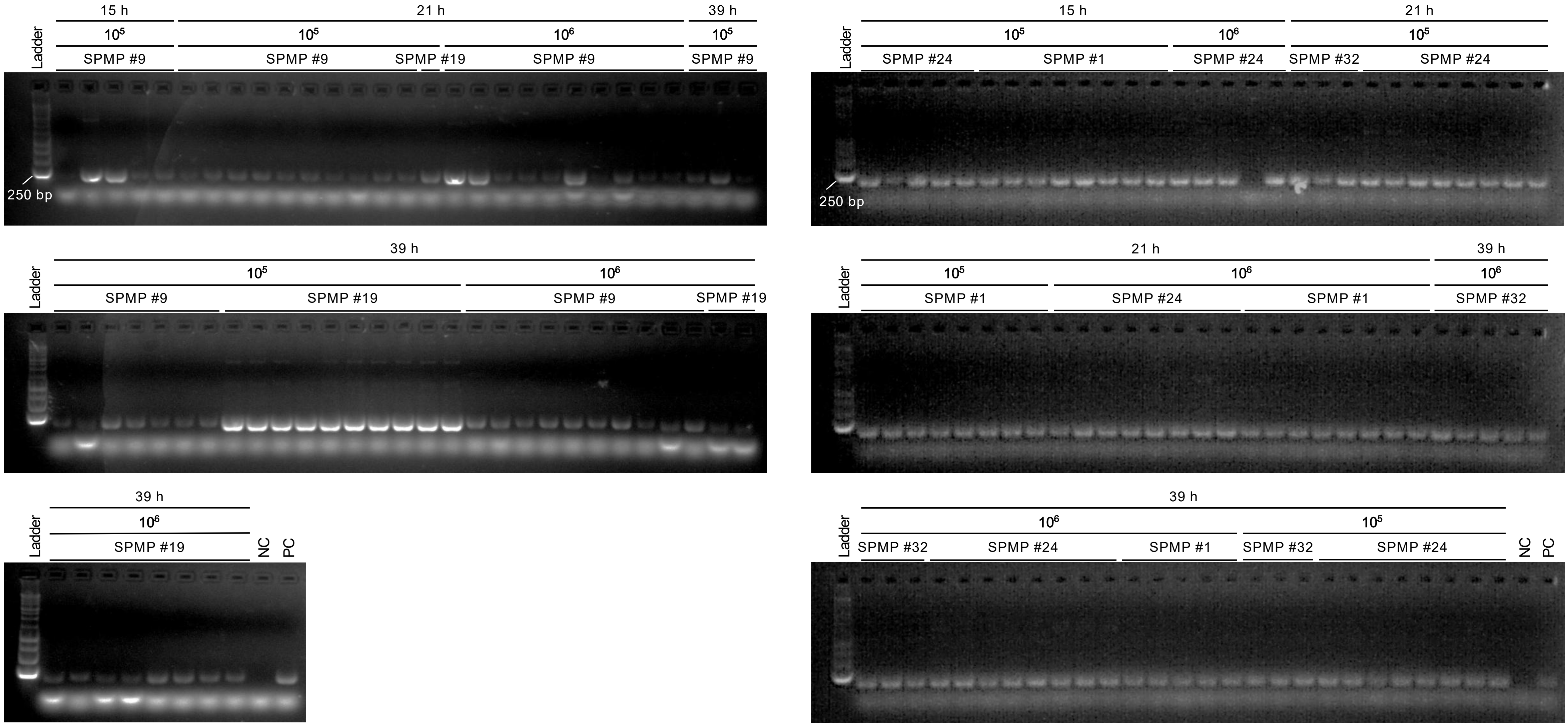

Supplementary Figure 4. Gel electrophoresis images for *xfp* gene PCR products from SPMP fecal samples. NC: negative control (nuclease-free water). PC: positive control (*B. adolescentis* pure culture).

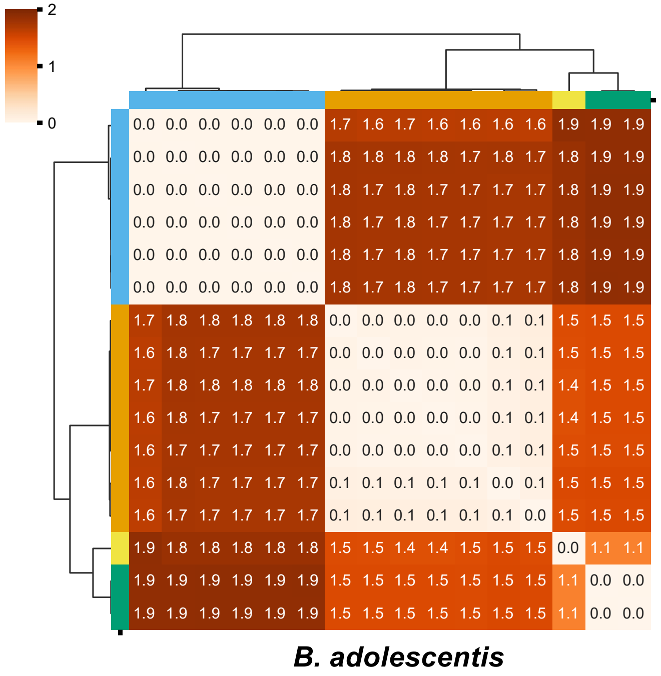

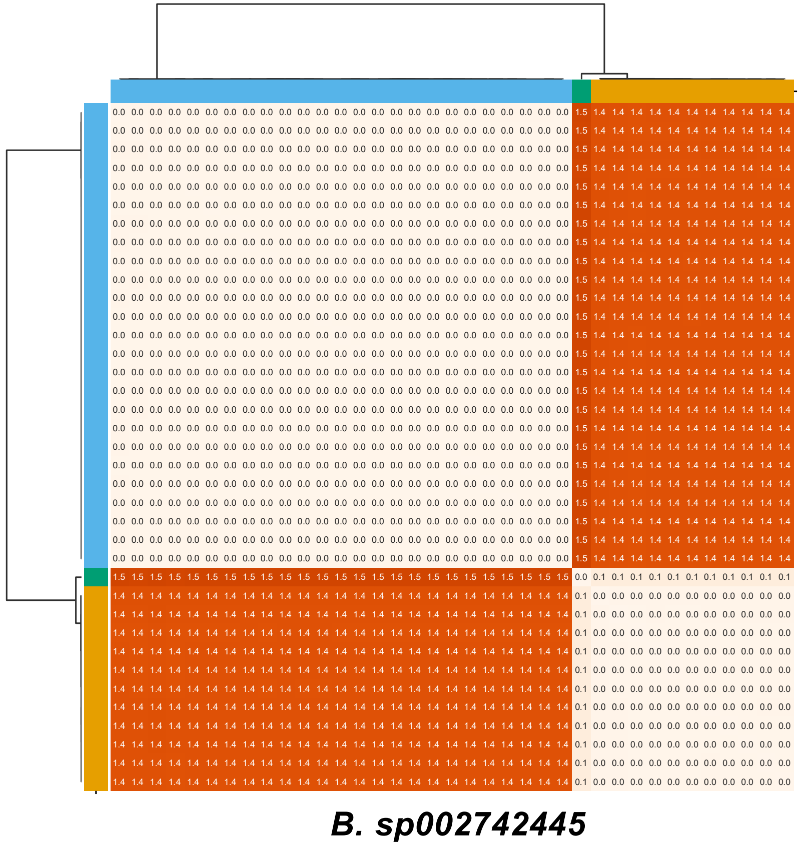

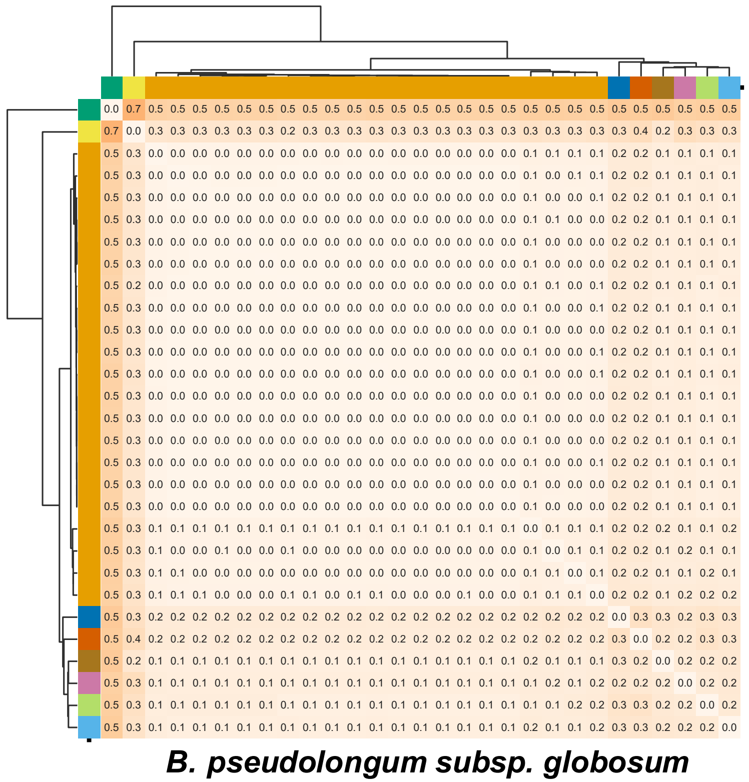

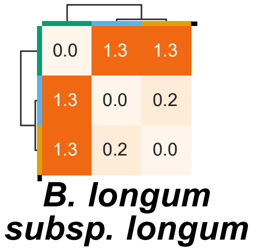

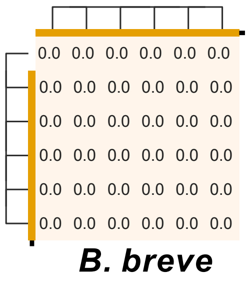

Supplementary Figure 5. Cluster maps showing pairwise ANI comparisons between SPMP isolates. Dendrograms were constructed using hierarchical clustering. External colors display isolates belonging to the same lineage (>99.9% ANI). Values shown are 1-ANI.

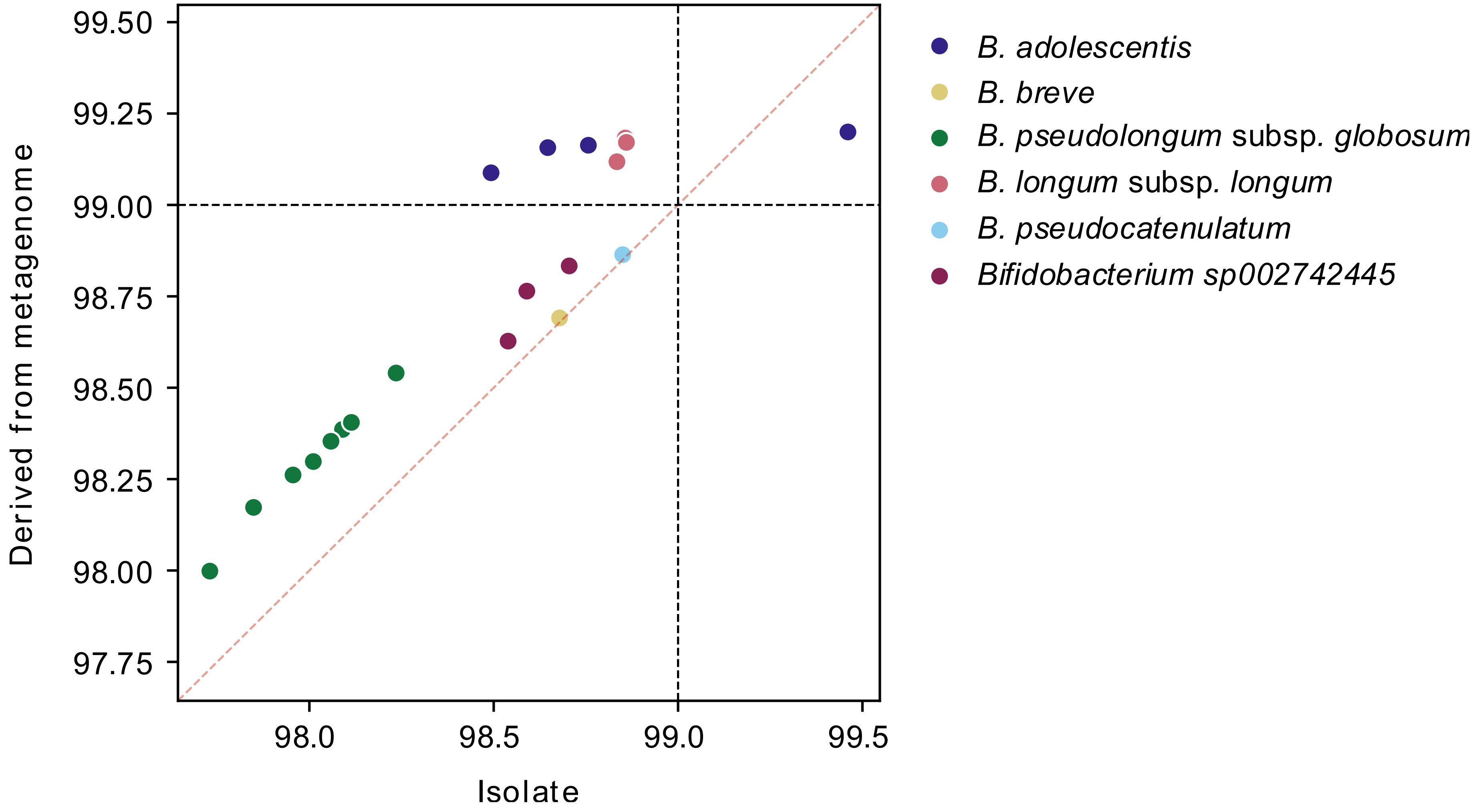

Supplementary Figure 6. Comparison of genomes obtained from cultures in this study with GTDB genomes. Each dot represents comparisons for a lineage-level representative from this study (clustered at 99.9% ANI). ANI values (obtained from SkANI) relative to most similar MAG and culture-based genomes (Isolate) in GTDB are reported here. Red dotted line represents the diagonal for visual aid. Black dotted line delimits 99% ANI.

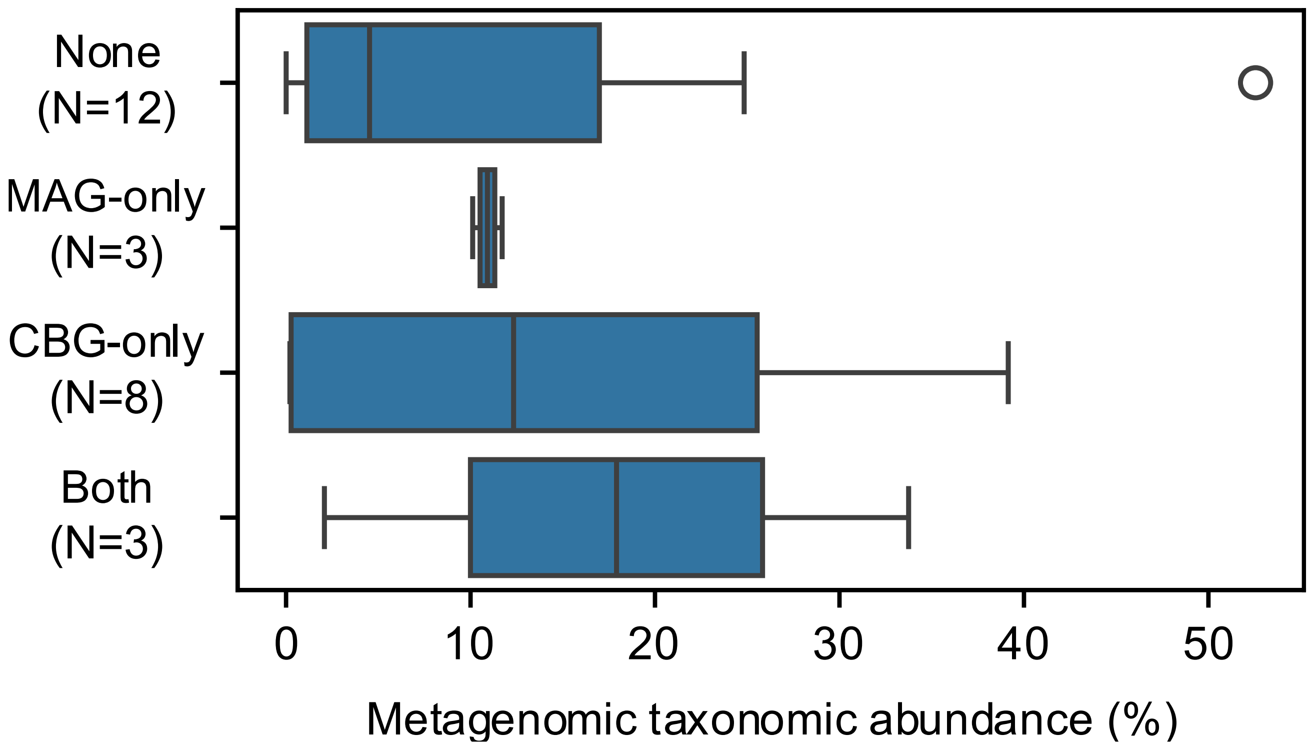

Supplementary Figure 7. Relative abundances of *Bifidobacterium* lineages in metagenomic data. MAG: metagenome-assembled genome from Gounot et al, 2022; CBG: culture-based genomes from this study. Both: lineages with both MAGs and CBGs. None: lineages for which medium- to high-quality genomes could not be assembled. Relative abundances are computed based on metagenomic data from Gounot et al, 2022 (n=109).

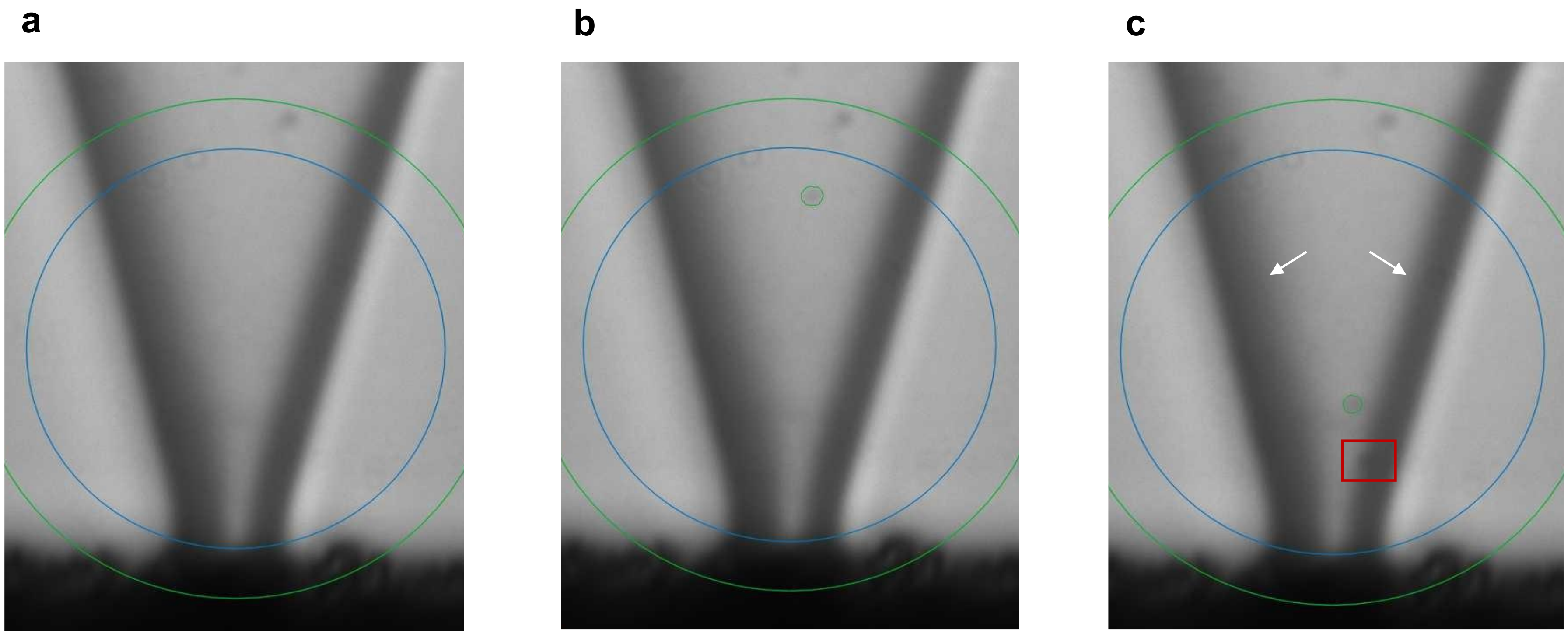

Supplementary Figure 8. B.SIGHT nozzle images of a cartridge during a run. (a) Empty cartridge. (b) A single cell detected and dispensed (circled in green). (c) Two cells (doublets) detected and dispensed (marked in green and red). Dark edges of the cartridge (white arrows) and low camera resolution could potentially obscure cells in the region of interest (ROI), leading to droplets with two or more cells.

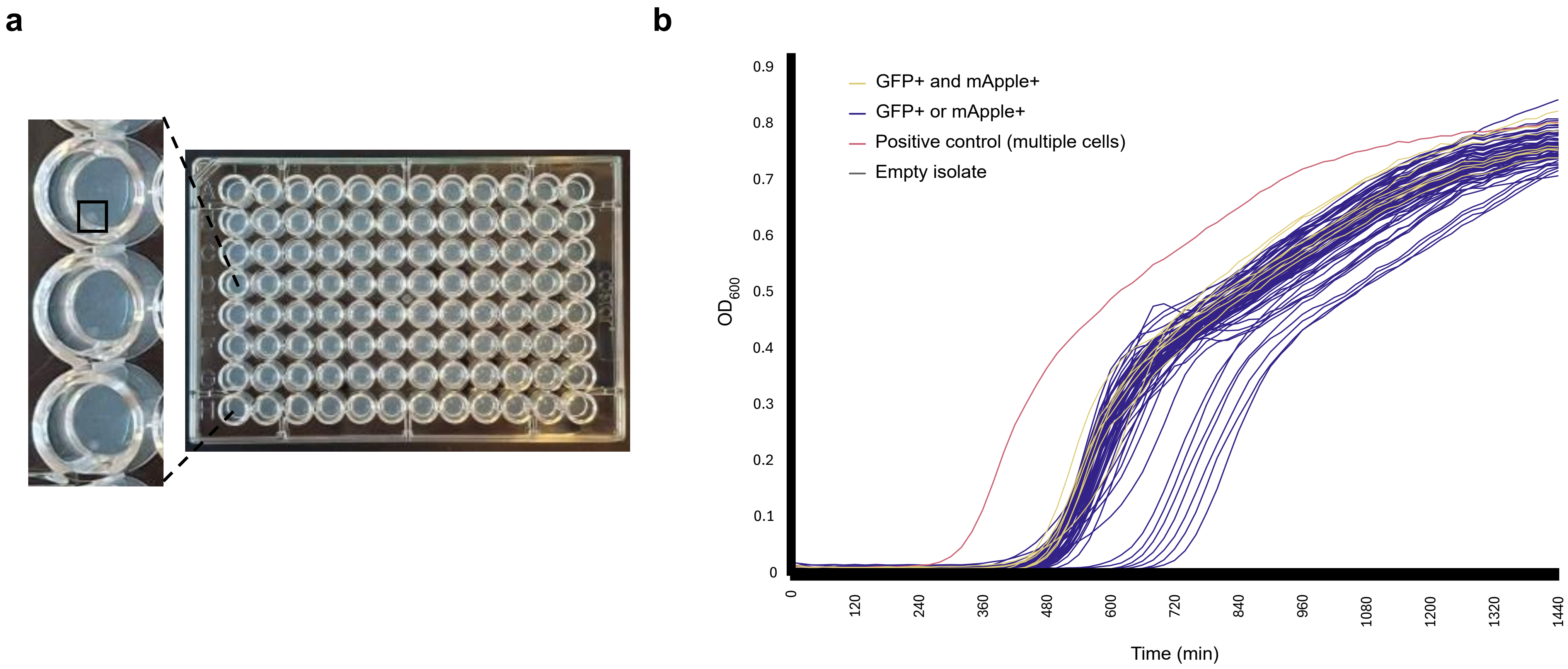

Supplementary Figure 9. Alternative methods were not useful for detecting doublets. (a) Visual inspection of colony growth on agar-filled plates. Double colonies could not be visibly discerned when cells were dispensed onto agar-filled wells. All colonies appear as the one shown in the black square. (b) Examination of growth curve variations over 24h. GFP+ and mApple+ doublets are indicated in yellow. Cultures with single fluorescence, GFP or mApple, are indicated in blue. Positive control (400 droplets) is indicated in red. Empty isolates with no observable growth are indicated in gray. There was no significant difference between growth curves of doublets (identified using dual fluorescence detection) and single cells.

a

Media		*E. faecium*	*B. breve*	*B. infantis*	Dispensed *Enterococcus* Isolate						
					1	2	3	4	5	6	7
Agar	BSM	+	+	+	+	+	+	+	+	+	+
	BSM-SUP	+	+	+	+	+	+	+	+	+	+
	BSM-MUP	-	+	+	-	-	-	-	-	-	-
Broth	BSM	+	+	+	+	+	+	+	+	+	+
	BSM-SUP	+	+	+	+	+	+	+	+	+	+
	BSM-MUP	-	+	+	-	-	-	-	-	-	-

b

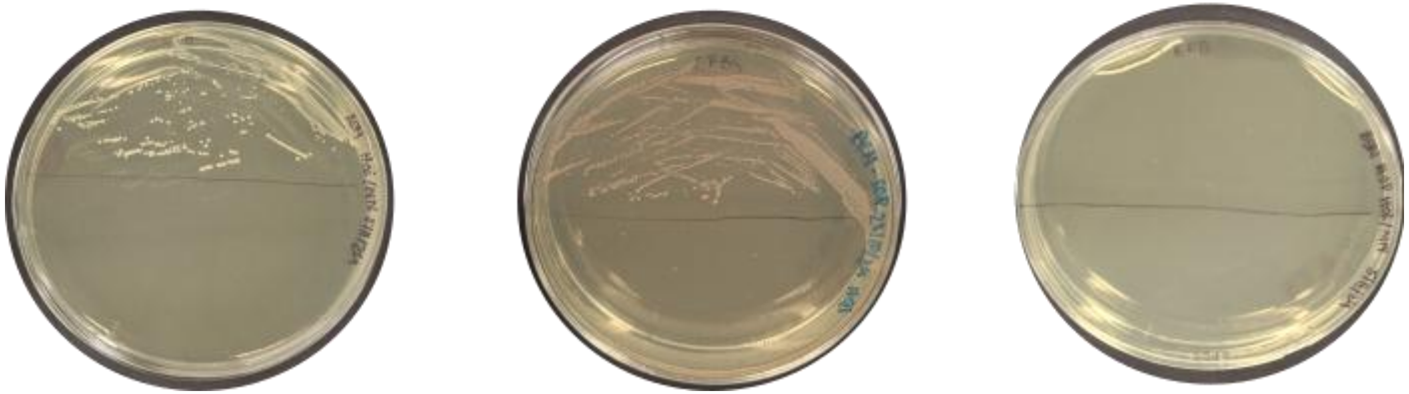

Supplementary Figure 10. Growth inhibition assay for *Enterococcus* and *Bifidobacterium* species using different BSM-based media. Isolates were obtained from stool sample and identified using 16S Sanger sequencing. (a) Growth of *E. faecium*, *B. breve* and *B. longum* subsp. *infantis* and dispensed isolates in BSM-MUP compared to BSM and BSM-SUP. “+” indicates growth after 48h. “-“ indicates growth inhibition after 48h. (b) Images of growth of *E. faecium* streaked on respective agar media. Only the top half of the agar plates were used for streaking.

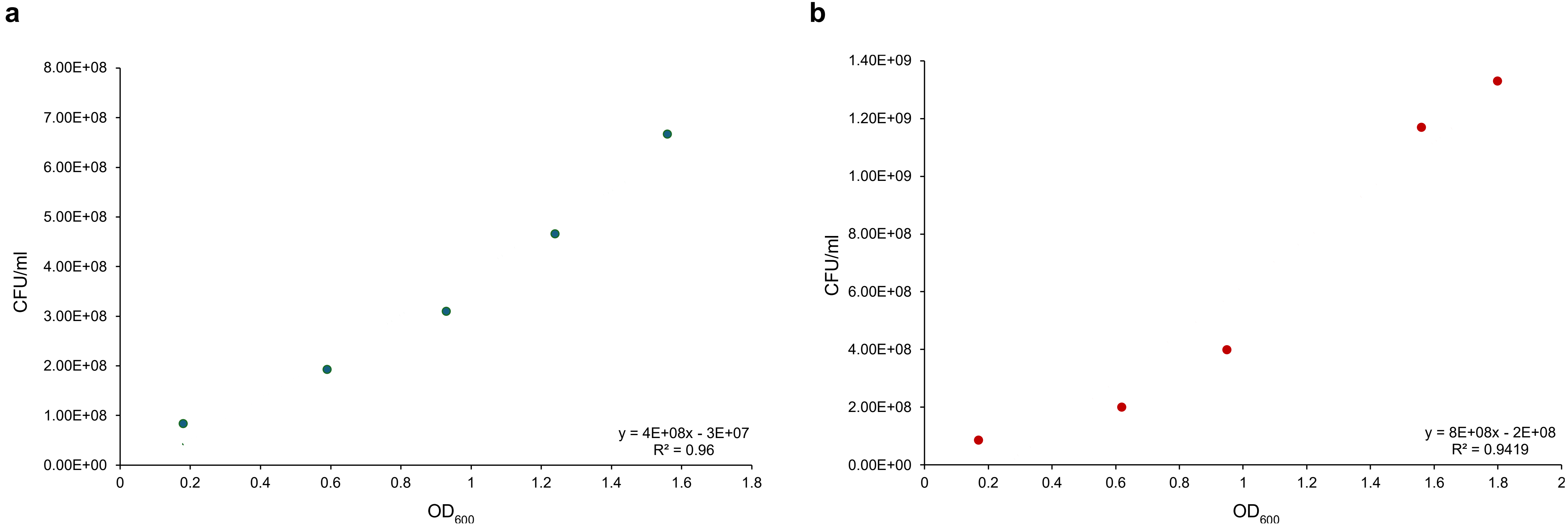

Supplementary Figure 11. OD_{600}-CFU relationship for the two fluorescent *K. pneumoniae* strains. Each dot represents a OD_{600}-CFU paired reading for (a) *K. pneumoniae* transformed with pKPC::sfGFP1 and (b) *K. pneumoniae* transformed with pKPC::mApple. The dotted lines are the best-fit lines, whose equations are show at the bottom right corner of each plot. R-squared values are also given at the bottom right corner of each plot.
